# Bridging landscape and perceived restorativeness: an empirical study of greenways along the Grand Canal, Hangzhou

**DOI:** 10.3389/fpsyg.2026.1742799

**Published:** 2026-02-05

**Authors:** Yue Wang, Mengjie Yang, Yijun Lu, Qiaoyi He, Youli Zhang, Qiaoqiao Wang, Bohao Wang, Xinmiao Ruan, Youjin Chen, Xinyue Zhang, Guofu Yang

**Affiliations:** 1 School of Art and Archaeology, Hangzhou City University, Hangzhou, China; 2 School of Medicine, Hangzhou City University, Hangzhou, China

**Keywords:** landscape ecological design, landscape ecological health, neural network modelling, perceived restorativeness, riverside greenway

## Abstract

**Context:**

Although numerous studies have shown that urban riverside greenways benefit mental health, the mechanisms through which landscape design, particularly ecological design, and influences psychological restoration remain underexplored.

**Objectives:**

This study addresses this gap by investigating the potential nonlinear effects of design elements (including plant design, ecological design, path design, and facility design across four dimensions and 17 indicators) on psychological restoration. Specifically, exploring the impact of various elements on psychological restoration and assesses the differences in the contributions of each design dimension to psychological restoration.

**Methods:**

This study focuses on the Hangzhou section of the Beijing-Hangzhou Grand Canal, tracking 1,052 questionnaires to collect data on design element evaluations and psychological restoration perceptions.

**Results:**

The results show that: (1) in landscape design, the combination of plant design, ecological design, and facility design significantly outperforms any single element or two-element combinations in terms of restorative effects, demonstrating a synergistic effect; (2) in terms of specific mechanisms, plant color, permeable pavements, and facility quality exhibit a U-shaped relationship with psychological restoration, while ecological revetments show an inverted U-shaped relationship; (3) vegetation coverage, plant color, and resting facilities are key factors in promoting psychological restoration.

**Conclusion:**

This study reveals the synergistic and nonlinear effects of landscape design in urban riverside greenways on psychological restoration, providing valuable design dosage references for urban planners and designers. It offers important insights for the future design of urban riverside greenways that harmonize landscape and human health.

## 1 Introduction

Perceived restorativeness, a concept rooted in Attention Restoration Theory (ART) (Kaplan and Kaplan, 1989), refers to the dynamic process by which individuals recover cognitive abilities, reduce mental fatigue, and experience positive psychological outcomes in environments characterized by qualities such as “being away, fascination, extension, and compatibility”(Hartig et al., 1997a). This restorative effect is recognized as a key mechanism through which landscape environments contribute to mental well-being (Hung, 2025). Urban blue–green spaces, as a complex artificial–natural composite ecosystems, enhance the comfort and diversity of urban landscapes (Bera et al., 2021) and foster the public’s perceived restorativeness (Zhou et al., 2023). Among these, urban riverside greenway, as an important type blue–green space, have attracted growing attention in urban health research due to their unique hydrophilic qualities, ecological connectivity, and high accessibility.

An urban riverside greenway is an ecological corridor that includes a variety of natural and artificial elements, with rivers and waterfront spaces as its natural substrate. As a linear space where land and water intersect, the riverside greenway plays a vital role in the urban public space and the urban ecosystem. From an ecological health perspective, urban riverside greenways have critical environmental functions (Zhang and Zhang, 2020), such as improving the surrounding microclimate, supporting biodiversity (Wang et al., 2024), and reducing air pollution (Nowak et al., 2006). Furthermore, research has shown that properly planned and designed urban riverside greenway environments are essential for promoting users’ perceived restorativeness, reducing stress (Shan et al., 2022), and enhancing mental health (Zhu et al., 2024). However, existing research has predominantly concentrated on the macro-level differences in the health benefits of urban riverside greenways as an environmental type (Cao et al., 2024; Li et al., 2022), and the mechanisms through which their design explicitily translates into actual health benefits have yet to be thoroughly explored.

In addition, differences in individuals’ social attributes often lead to significant variations in their perceived need for restorative environments (Hoyle et al., 2017). Multiple studies have confirmed that even the same green space or park can exhibit significant differences in restorability between groups with different social attributes (Arnberger et al., 2024). Yin et al. (2025) note that these differences also lead to spatial exclusion and even exacerbate inter-group conflicts. Urban riverside greenways, as important public spaces that accommodate diverse social groups, must be designed to precisely respond to these group differences. Exploring the perceived differences and functional expectations of different demographic groups for riverside greenways is not only key to enhancing environmental restorative effectiveness but also an important path to achieving urban spatial justice and environmental equity (Dinnie et al., 2013).

The planning and design of urban riverside greenways can generally be understood through four dimensions: paths, plantings, facility, and ecological design (Ministry of Housing and Urban–Rural Development, 2016; Cao et al., 2024). Among these, path design serves as the structural backbone of the greenway system. By enhancing spatial continuity and accessibility, it creates a safe and user-friendly network for slow mobility, such as walking and cycling. Plant design focuses on design elements such as riparian planting zones and buffer zone vegetation communities. These elements help maintain ecological health by promoting biodiversity and regulating the microclimate. In addition, they play a significant role in enhancing users’ psychological well-being through seasonal variations and diversity in design (Hao et al., 2024a). Facility design includes elements such as seating areas, pavilions, and signage systems. These features fulfill multifunctional needs while also helping to reduce users’ mental stress (Van Herzele and Wiedemann, 2003). Meanwhile, ecological design—an essential component of the urban ecosystem—aims to integrate ecological functionality and sustainability into the built environment. Through the application of ecological technologies (e.g., ecological revement, permeable pavement, and ecological floating islands), it enhances both the resilience and multifunctionality of riverside spaces. Research has indicated that incorporating eco-design into urban green spaces can significantly improve landscape ecological health. Taking ecological revetment as an example, they play a crucial role in urban river ecological restoration by filtering surface runoff, enhancing water self-purification capacity, and providing habitats for plants and animals (Wang et al., 2024). In addition to their environmental benefits, natural elements have also been widely recognized for enhancing perceived restorativeness—by visually and physically embedding natural features into urban environments (Carrus et al., 2015; Hung, 2025). Despite the widespread application of ecological design in riverside greenways and its recognized benefits for landscape ecological health, the complex mechanisms between ecological design and perceived restorativeness have not been fully explored.

To analyze the potential relationship between planning and design and Perceived restorativeness. Previous studies have predominantly used linear models to examine the relationship between the intensity of exposure (i.e., “dose”) to urban blue–green spaces and health outcomes (i.e., “response”), leading to a widespread belief that “‘greener is always better for mental health’” (Cox et al., 2017; Van Den Berg et al., 2010). However, the impact of urban blue–green spaces on mental health is not always positive. It is essential to consider the potential negative effects of natural environments, such as how overly dense vegetation can induce feelings of claustrophobia, leading to discomfort among users (Fan et al., 2020). In recent years, a growing body of empirical research has suggested that the relationship between urban green spaces and mental health may be more complex and non-linear, following a “dose–response” pattern (Jiang et al., 2025). According to this perspective, psychological benefits increase rapidly within a certain range of green space exposure, but beyond a certain threshold, these benefits may level off, or even decrease, potentially leading to negative effects (Zhao et al., 2025). In the context of rapid urbanization, the continuous growth in population and building density places significant constraints on the expansion of urban blue–green spaces. As a result, there is an urgent need to optimize landscape design within the limited space of riverside greenways to create the most restorative environments (Wolch et al., 2014). Therefore, an important scientific challenge in the field of environmental restoration is to uncover the potential nonlinear “dose–response” curve between riverside greenway design features and perceived restorative health benefits. Additionally, identifying the “optimal benefit threshold” of greenway design has become a critical focus for future research.

However, traditional studies have mostly relied on linear models such as multiple linear regression, which are limited in capturing the complex nonlinear relationship between urban riverside greenways and perceived restorative benefits. Therefore, this study introduces the Multi-Layer Perceptron (MLP), a type of neural network model, to better capture the intricate relationship between design factors and perceived restorative outcomes (Lindner et al., 2015). The Multi-Layer Perceptron (“black-box” nature of machine learning models), this study further incorporates the SHAP (SHapley Additive Explanations) model, which is rooted in game theory. As a key tool in the field of explainable artificial intelligence (XAI), SHAP enables effective quantification of each feature variable’s contribution to the model output (Moncada-Torres et al., 2021; Yang et al., 2024).

This study employs a cross-sectional design to examine the impact of urban greenways on immediate mental health recovery. We constructed a subjective landscape perception index system for riverside greenways to measure greenway landscape quality, collected data from user questionnaires to assess the difference in perceived recovery before and after greenway use, and applied an interpretable machine learning algorithm (MLP combined with SHAP) to explore the mechanisms by which key design factors influence the perceived restorative benefits of riverside greenways. The focus was on the following questions: (1) Which design features of urban riverside greenways are primary drivers that influence residents’ perceived restoration? (2) What are the mechanisms by which these key design features influence perceived restoration? (3) How do these design features play a differentiating role in the perceived restoration of respondent groups with different demographic characteristics? The results of this study can provide a scientific basis for optimising the planning and design of urban riverside greenways and achieving a “win-win” situation for both ecological health and human health.

## 2 Materials and methods

### 2.1 Research objects

**Figure 1:**
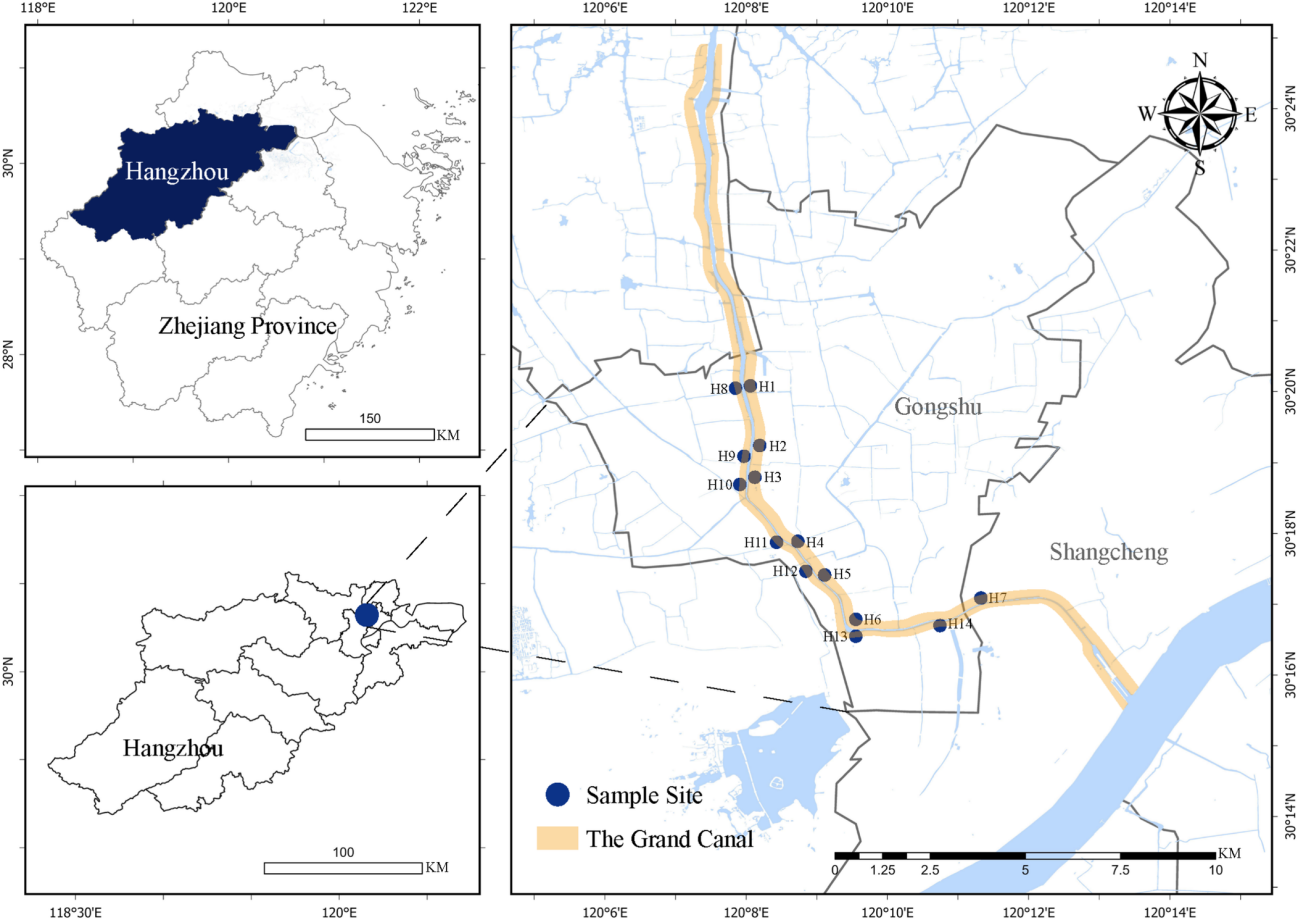
Distribution of research sample sites.

Hangzhou, located in eastern China, is characterized by a dense river network. In recent years, Hangzhou has developed an extensive system of riverside greenways, which now account for 36% of the total length of all greenways in the city, making them the most prevalent type of greenway (Hangzhou Bureau of Statistics, 2023). One of the most significant water systems in the city is the Beijing-Hangzhou Grand Canal, which stretches approximately 37 kilometers through the urban core. This section of the canal runs through a high-density area, with its banks featuring a variety of land uses, including residential neighborhoods, schools, commercial districts, and public spaces, within a watershed area of about 726.6 km^2^ (Figure 1). It exhibits typical linear public space characteristics in terms of spatial structure. Over 70% of its sections are relatively narrow, with locally dense vegetation along the route, and it has generally formed a material environmental foundation dominated by slow travel and lingering (Supplementary Figure 1). The canal provides local residents with abundant linear waterfront public spaces and high-quality landscape corridors. Given these characteristics, the Hangzhou section of the Grand Canal serves as a typical case for studying the relationship between urban riverside greenways and residents’ health. As such, this study focuses on the Hangzhou section of the Beijing-Hangzhou Grand Canal, which holds significant research value. It offers direct scientific support for refining the design and enhancing the health benefits of Hangzhou’s riverside greenways. And providing empirical data and design guidelines that can inform the planning and development of riverside greenways along the Grand Canal and in other similar high-density urban areas across the country.

### 2.2 Sample point setting and questionnaire

#### 2.2.1 Sample point selection

**Figure 2:**
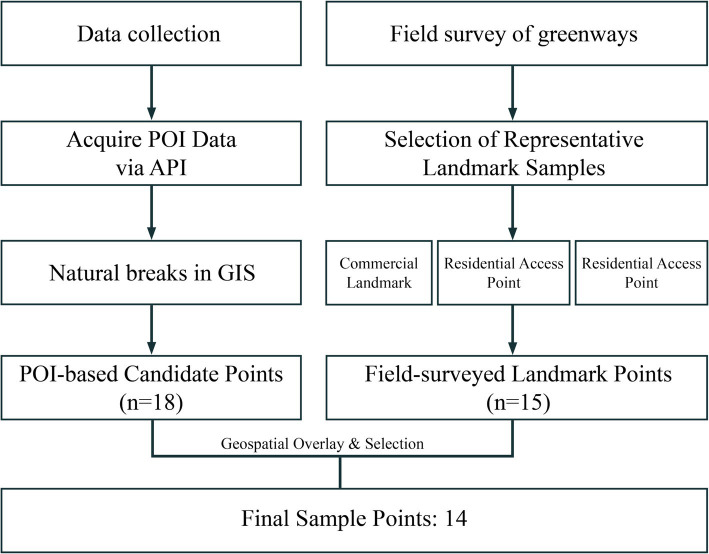
Flowchart of sample point selection.

The sample point method was used for data collection (Xu et al., 2022). Firstly, the POI data of the greenway on the east and west sides of the Grand Canal were obtained through the Gaode Open Platform using Python tools; secondly, the natural breakpoint classification was carried out by combining the POI data, and in order to cover as many numbers of the Grand Canal greenway user groups as possible, 14 survey sample points were selected along the Grand Canal Greenway based on the vitality points with the highest pedestrian flow. The selected sample points are mainly commercial landmarks, community entrances and exits and traffic facilities coverage points, and the length of the greenway covered by the survey is about 14 km, covering 33.4% of the total length of the study area (Figure 2).

#### 2.2.2 Questionnaire design and survey

To ensure the rationality of questionnaire design and data validity, this study conducted a pre-survey. The survey targeted users of landmark sample points along the riverside greenway of the Beijing-Hangzhou Grand Canal in Hangzhou, with a sample size of 80 respondents. The questionnaire covered basic user information (gender, age, purpose of use, etc.), a design rating scale, and a restorative qualities scale. Preliminary data indicated that the questionnaire took 5 to 8 min to complete, with over 90% of respondents using the greenway primarily for leisure activities, while exercise or commuting purposes were less common. Based on these findings, the questionnaire underwent content optimization: certain questions were removed or rephrased, while ensuring the completion time remained within the range observed in the preliminary study. This refinement aimed to enhance both the efficiency and accuracy of data collection.

. The formal experimental questionnaire for this study comprises three sections: basic information, design factor rating scale, and restorative rating scale. Basic information includes demographic characteristics such as “gender” and “age.” The design factor rating scale comprises 17 indicators, including “plant species” and “ecological revetment,” to understand respondents’ perceived environmental restorative experiences during greenway use. The restorative rating scale consists of pre-measurement and post-measurement forms to assess individuals’ perceived restorative levels before entering and after leaving the greenway. The questionnaire was administered in two groups: one to individuals entering the greenway, comprising basic information and the pre-recovery measurement scale; the other to those exiting, including basic information, the design factor rating scale, and the post-recovery measurement scale. Differentiated scale content prevented measurement bias from repetition. All scales employed a 1–7 point rating system (1 = very weak, 4 = moderate, 7 = very strong) to indicate intensity levels.

#### 2.2.3 Sampling survey

In this study, we adhered to ecostatistics principles by selecting a sampling survey design based on observational experiments. Following the optimization principles for observational experiments in statistics (Gotelli and Ellison, 2004), we conducted repeated sampling at multiple representative entry/exit points and employed a large sample size to mitigate the impact of individual variation.

First, to ensure statistical power (Cohen, 1988), a pre-trial sample size calculation was performed using G*Power 3.1 software. Parameters were set as follows: significance level (*α*) = 0.05, statistical power (1-*β*) = 0.95, effect size *f*^2^ = 0.15, and number of predictors = 17. The calculation indicated a minimum required sample size of 208 individuals.

The experiment was conducted from December 2024 to January 2025. The observation periods were selected during clear weather with cloud cover <30%, temperatures between 5 and 10 °C, and Beaufort wind force 2–3, specifically from 9:00 to 11:00 and 13:00 to 15:00. The survey process comprised three steps: (1) Conduct crowd surveys at 14 high-traffic sample points designated as entry/exit points. Respondents were interviewed; those entering for less than 10 min were classified as incoming crowds, while those preparing to leave or exiting within 10 min were classified as outgoing crowds (Tu et al., 2026). (2) After explaining the study objectives and obtaining informed consent, participants completed questionnaires. For those completing post-test questionnaires, the landscape design factor rating scale was explained in conjunction with the greenway environment to ensure accurate understanding and objective scoring. (3) Researchers reviewed questionnaires for omissions. This study collected 1,160 raw questionnaires (573 entry questionnaires, 587 exit questionnaires). Subsequently, initial questionnaires underwent rigorous multi-stage screening using post-identification methods summarized from existing research (Zhong et al., 2021). Three primary methods were employed: embedded identification scales, response pattern recognition, and reaction time analysis (detailed methods are provided in the Supplementary Material). Ultimately, 1,052 valid questionnaires were obtained (a validity rate of 90.34%), with 526 each in the entry group and exit group.

### 2.3 Research methods

#### 2.3.1 Indicator system of design factors affecting the resilience of riparian greenways

Based on a comprehensive review of existing literature (Xu et al., 2024), environmental characteristics that have been confirmed to be related to the perceived restoration effects of riverside greenways were identified and summarized. In addition, expert opinions from fields such as landscape design, environmental psychology, and public health were incorporated. From this, 25 potential indicators were initially selected. These indicators were further refined through preliminary surveys and interviews with the public, ultimately resulting in the identification of 17 design factors across four dimensions: Plant design, Ecological design, Path design, and Facility design (Supplementary Table 1). This process led to the development of an indicator system to measure the perceived restorative capacity of urban riverside greenways. The Natural-based system dimension includes Plant design and Ecological design, while the artificial system dimension includes Path design and Facility design. Additionally, the results showed that Cronbach’s alpha values for all indicators were greater than 0.8, indicating high reliability. The KMO value was 0.912, and the data passed the Bartlett sphericity test (*p* < 0.001), confirming both the credibility and validity of the data, and supporting the feasibility of further analysis. The Perceived Recovery Capacity Assessment Scale for Riverside Greenways developed in this study incorporates a degree of subjectivity. However, through rigorous investigator training, dual cross-scoring, and consistency verification, we have effectively enhanced the reliability and validity of the data.

#### 2.3.2 Restorativeness scale

In order to provide a subjective evaluation of the restorativeness of the environment, researchers initially developed the Perceived Restorativeness Scale (PRS) (Hartig et al., 1997b). Due to the large sample size collected in this study, a large number of PRS scale items was not appropriate, so the short version of The short-version revised restoration scale (SRRS) developed by Han (2003) was used in this study. The SRRS has four restorative dimensions, Emotion, Physical, Preference, Action, with two items for each dimension, for a total of eight items. In this study, the specific questions of the SRRS scale were appropriately adapted according to the environmental characteristics of the riverside greenway (Supplementary Tables 2, 3). The scale subsequently passed reliability (Cronbach’s alpha = 0.528) and validity (KMO = 0.652, Bartlett’s test *p* < 0.05) tests, which indicates that it has a certain degree of consistency and validity in the measurement process.

#### 2.3.3 Interpretable machine learning model based on multilayer perceptron

A multilayer perceptron (MLP) is a classical feed-forward artificial neural network (Hecht-Nielsen, 1987) whose core structure consists of an input layer, a hidden layer and an output layer. The input layer receives feature variables (environmental indicators), the hidden layer passes the information layer by layer through a nonlinear activation function (ReLU), and finally the output layer generates the prediction results. The SHapley Additive exPlanations (SHAP) method is also combined to quantify feature contributions and parse decision paths to enhance the interpretability of the model.

Based on 1,052 questionnaires from riverside greenway users, this study constructed a multi-layer perceptron (MLP) regression model. Seventeen design factor indicators—including vegetation coverage, permeable paving, and facility comfort—served as input features, while the composite perceived recovery score acted as the dependent variable. To ensure model robustness, multicollinearity diagnostics were performed prior to modeling. Results indicated that (Supplementary Table 4) the absolute values of correlation coefficients between all variables were below 0.8, and the Variance Inflation Factor (VIF) values for all variables were less than 5, confirming the absence of high linear correlation among independent variables.

Simultaneously, hyperparameter optimization (Supplementary Table 5) was conducted for the MLP regression model: features were standardized and split into training and test sets at the default 8:2 ratio. Manual exploration was performed across activation functions (ReLU/Tanh), regularization coefficients (0.0001–0.01), learning rates (0.0001–0.01), batch size (32/64/128), and other core hyperparameters. The optimal parameter combination was selected based on the *R*^2^ metric. After validating the model’s generalization capability on the test set, the optimal model was applied to subsequent processes such as SHAP feature importance analysis and partial dependency graphing. This ensured the reliability of the model’s prediction results and the accuracy of the feature analysis conclusions. The model achieved a coefficient of determination *R*^2^ of 0.417 on the test set, indicating its predictive capability for perceived recovery benefits. To decipher the decision logic of the MLP model, the SHAP package in Python was employed to construct an explanatory framework. By calculating SHAP values for each environmental indicator, their marginal contribution to the perceived recovery score was quantified. Feature importance rankings and partial dependency plots were generated, revealing the complex nonlinear effects and threshold responses of individual design factors on perceived recovery.

#### 2.3.4 Data statistics and analyses

This study utilized SPSS 27.0 to process the data, filling in missing and extreme values with the median. Comprehensive perceived restorativeness scores for each sub-dimension of the short-version revised restoration scale (SRRS) were calculated using the mean value method. These scores were then normalized. Descriptive statistical analyses of demographic characteristics were performed using chi-square tests and analysis of variance (ANOVA). To assess the differences perceived restorativeness across the dimensions of the recovery scale, the Wilcoxon Signed-Rank Test was applied. The objective weights of the environmental indicators were calculated using the Entropy Weight Method (EMW), and these weights were then integrated with TOPSIS to calculate the overall evaluation score for each design dimension of the environmental indicators. Furthermore, based on redundancy analysis (RDA) within Canonical Analysis (CA), the R package “rdacca.hp” (Liu et al., 2023) was used, introducing the concept of hierarchical partitioning (HP) to quantitatively evaluate the contribution of each design dimension to the perceived restorativeness of urban riverside greenways.

## 3 Results

### 3.1 Descriptive statistical analysis

#### 3.1.1 Descriptive statistical analysis of respondents’ demographic factors

**Figure 3:**
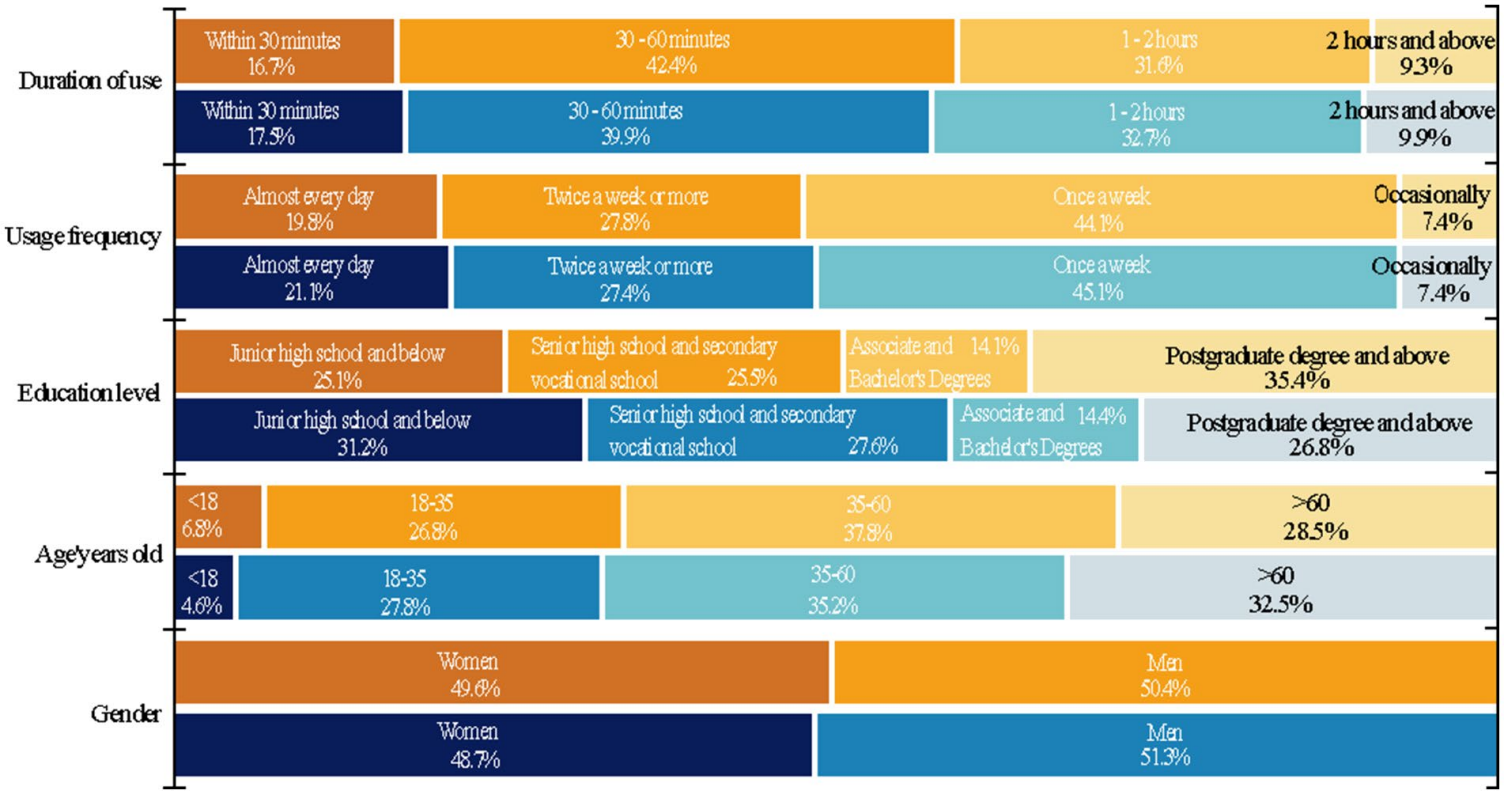
Descriptive analysis of respondents’ demographic factors. The yellow group indicates entering the crowd, while the blue group indicates leaving the crowd.

We compared the distribution of basic demographic characteristics—including gender, age, and educational attainment—between the two groups using a chi-square test (Supplementary Table 6) and confirmed no significant differences, indicating comparability between the two cohorts. Figure 3 displays respondents’ demographic information. (1) The proportion of females and males is 50.9 and 49.2%, respectively. (2) In terms of age distribution, the proportion of people under the age of 18 is 4.6%, the proportion of people between the ages of 18–35 is 27.8%, the proportion of people between the ages of 35–60 is 35.2%, and the proportion of people aged over 60 is 32.5%. (3) In terms of education level, it is mainly people with specialist, undergraduate and above education, accounting for 52.1%. (4) The usage frequency is mainly people who use it more than twice a week, accounting for 54.8%, and the duration of use is most popular among people who use it for 30 min-2 h (73.3%), with less people using it for less than 30 min and more than 2 h.

#### 3.1.2 Analysis of respondents’ frequency and duration of use on perceptual restorability effects

**Figure 4:**
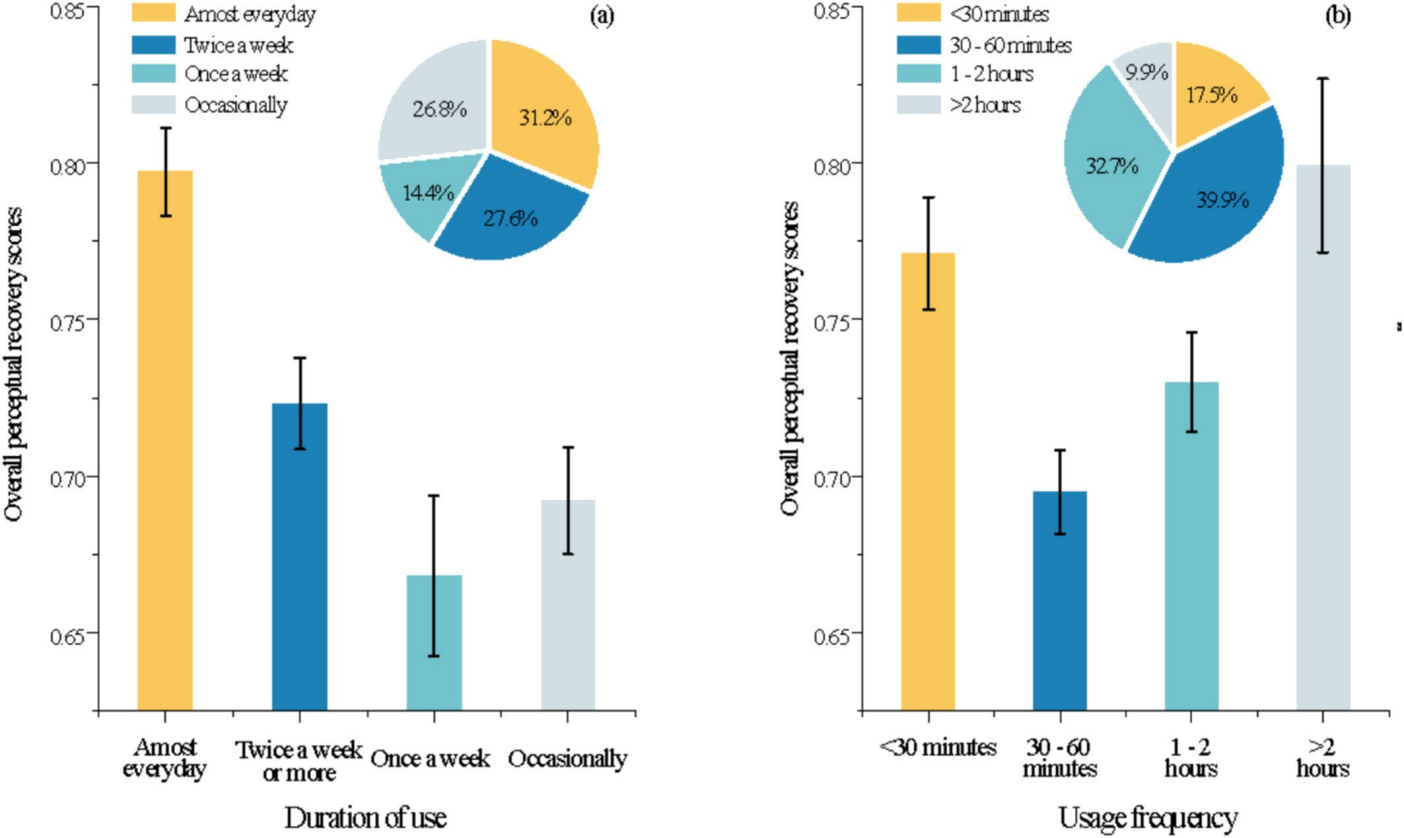
Difference analysis of respondents’ overall perceived recovery scores in terms of usage frequency **(a)** versus duration of use **(b)**. The pie charts indicate the percentage distribution of the number of people with different frequency of use versus the number of people with different duration of use.

A two-way ANOVA (Supplementary Table 7) revealed significant differences in perceived recovery levels among respondent groups before and after greenway use, across varying usage frequencies and durations (Figure 4). Among these, “nearly daily users” demonstrated significantly superior recovery effects compared to other groups, with recovery benefits gradually diminishing as usage frequency decreased (Figure 4a). Perceived recovery among users spending up to 30 min was slightly higher than among those using the greenway for 30–60 min. Beyond 1 h, recovery effects increased gradually with longer usage durations (Figure 4b).

#### 3.1.3 Differences in perceived restorativeness before and after greenway use

**Figure 5:**
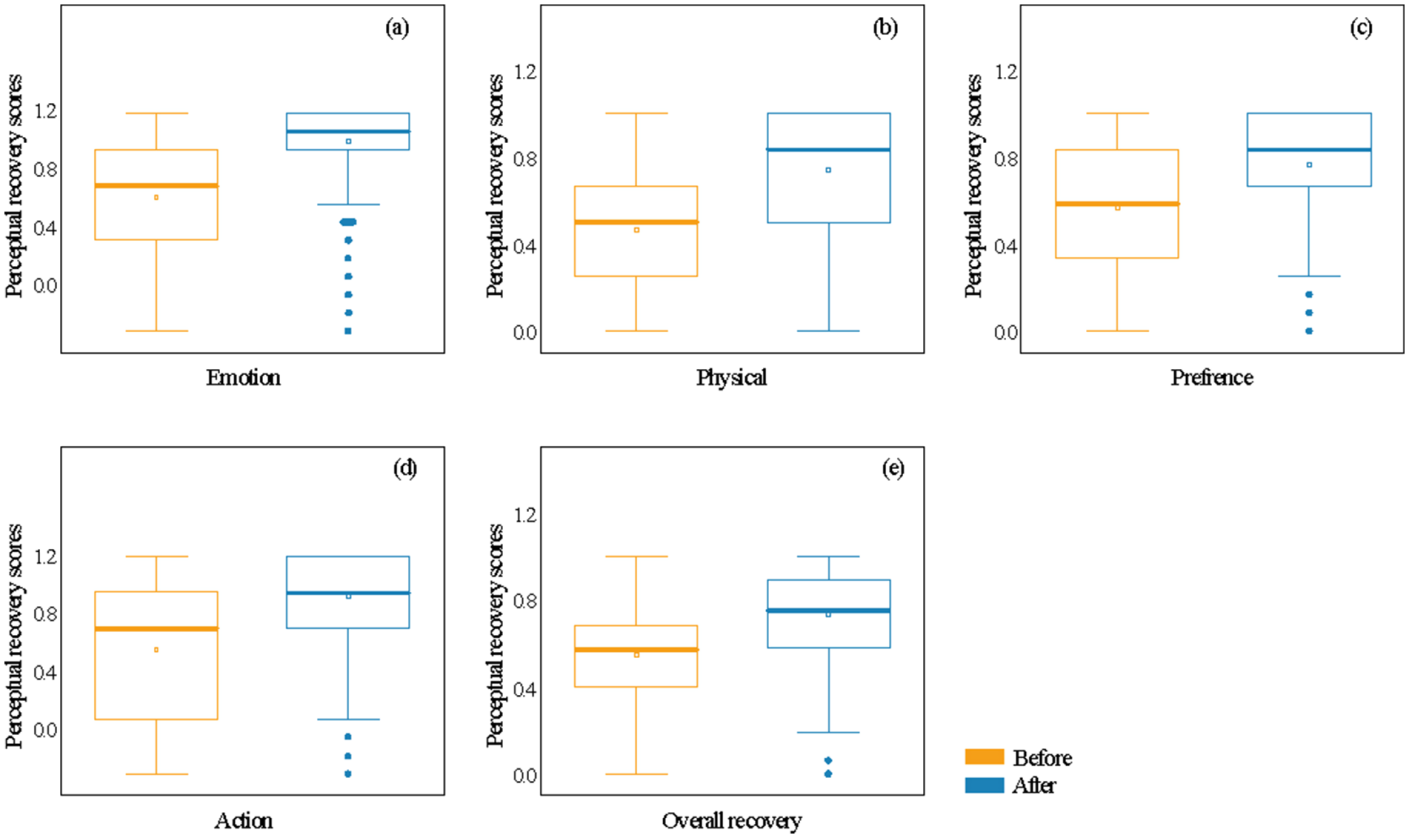
Differences in perceived recovery in emotional **(a)**, physical **(b)**, preference **(c)**, action **(d)**, and overall **(e)** recovery dimensions before and after respondents’ use of the greenway.

Results (Supplementary Table 8) indicate that individuals who left the crowd exhibited significantly greater perceived recovery in emotional, physiological, preference, behavioral, and overall recovery dimensions after using the greenway compared to those who entered the crowd (*p* < 0.001). Specifically, perceived restorative scores after using the greenway were higher than those before use in the Emotion (Figure 5a), Physical (Figure 5b), Preference (Figure 5c), Action (Figure 5d), and Overall restoration (Figure 5e) dimensions. This indicates a significant improvement in perceived restoration following greenway use. However, the degree of restoration in the behavioral dimension was relatively weaker. These findings suggest that urban riverside greenways provide notable restorative benefits, particularly in terms of Emotion, Physical, Preference, and Overall perceptions of recovery.

### 3.2 Comparison of perceived restorative effects across design dimensions

**Figure 6:**
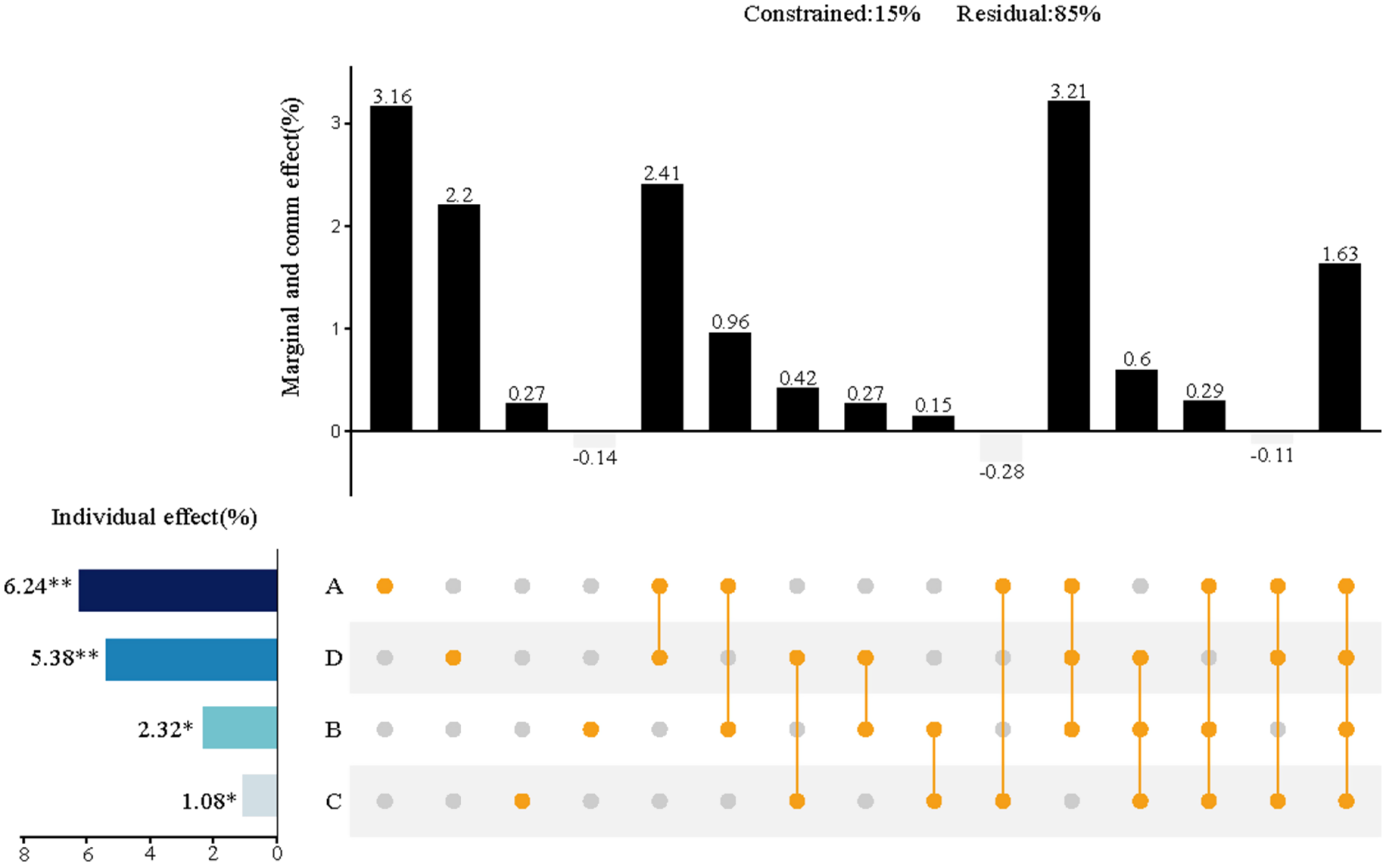
Relative importance of the four indicator dimensions (A Plant, B Ecology, C Path, D Facility) on the impact of mental health restoration of the urban riverside greenway. The results of variance decomposition and hierarchical segmentation analyses are presented using UpSet plots. In the dot plot on the right, each row corresponds to a criterion level factor. Isolated black dots in each column indicate the marginal effect of each factor, lines between multiple dots indicate the common effect of these factors, and the percentage of variance explained by each component (from the variance decomposition) is shown in the upper bar chart. Individual effects of each environmental factor are shown in the left bar graph. *p < 0.05; **p < 0.001.

The redundancy analysis shows that approximately 15% of the total variance in the perceived restorative benefits of the urban riverside greenway can be attributed to four design dimensions (Figure 6). Among these, Plant design (A) and Facility design (D) were the most influential, accounting for 6.2 and 5.4% of the variance, respectively. Although Ecological design (B) and Pathway design (C) contributed less—2.3 and 1.1%, respectively—each dimension still demonstrated a statistically significant individual effect. When examining the combined (interaction) effects across dimensions, the largest shared contribution came from planting design, ecological design, and facility design, which together explained 3.2% of the variance.

**Figure 7:**
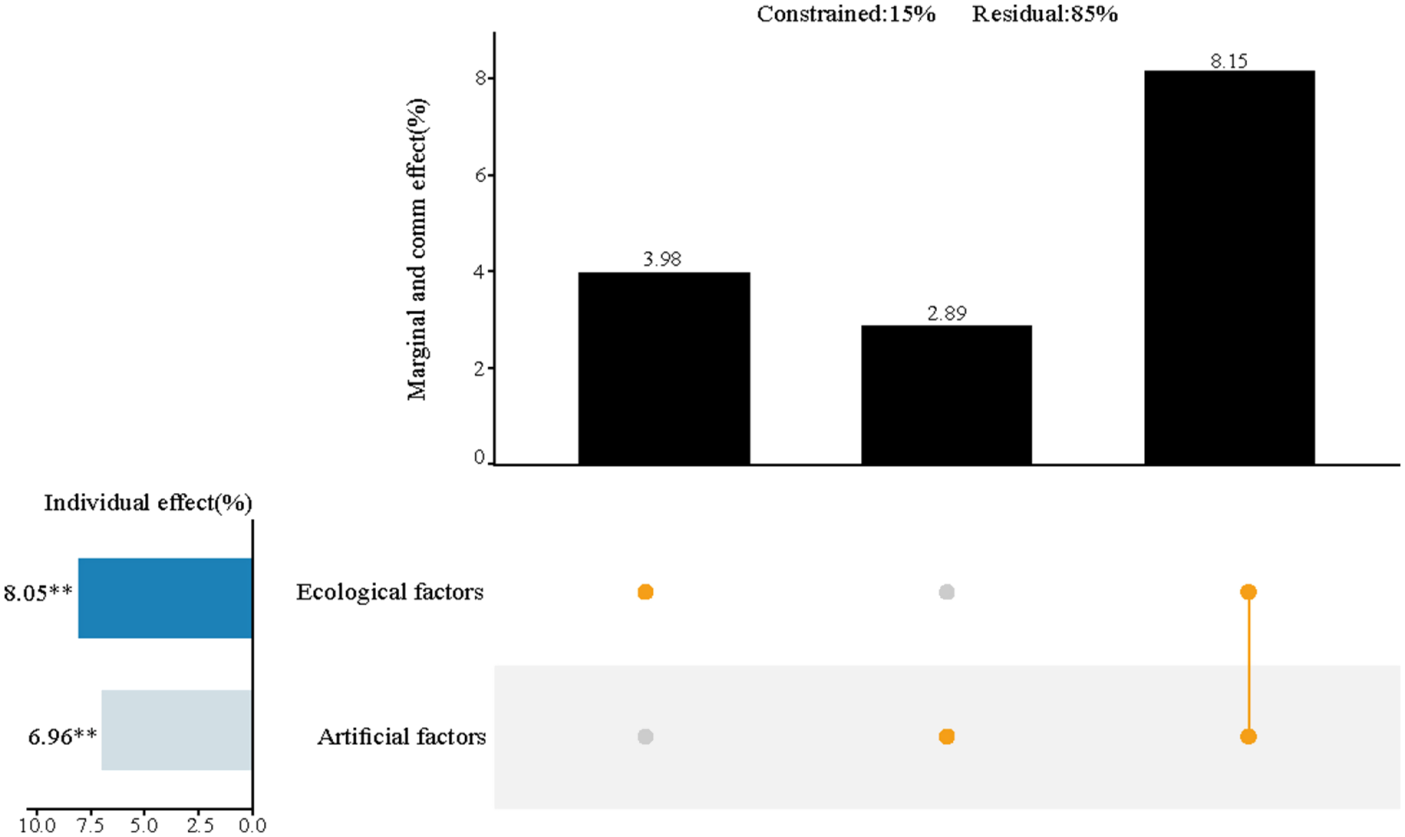
Relative importance of nature-based systems and artificial systems on the impact of mental health restoration of urban riverside greenways. The results of variance decomposition and hierarchical segmentation analyses are presented using UpSet plots. In the dot plot on the right, the three columns indicate the marginal effects of nature-based systems and artificial systems and the joint effect of the two (from variance decomposition), respectively. The left-hand columns indicate the separate effects of the two (from hierarchical partitioning). *p < 0.05; **p < 0.01.

In a broader comparison between Nature-based systems and Artificial systems (Figure 7), the Nature-based design dimension showed an importance of 8.1%, while the Artificial design dimension contributed 7.0%. The combined effect of both dimensions accounted for an additional 8.2% of the variance in perceived restorative outcomes.

### 3.3 Analysis of influencing factors

#### 3.3.1 Influence factor analysis of each age group

To explore the differences in the restorative effects of landscape elements across various age groups, this study develops four perceptual restoration models using an MLP combined with SHAP methods. These models correspond to four age groups: 0–18 year’s old, 18–35 year’s old, 35–60 year’s old, and 60 year’s old and above. Additionally, SHAP Beeswarm plots are generated to visually analyze the importance of different landscape elements for each age group.

**Figure 8:**
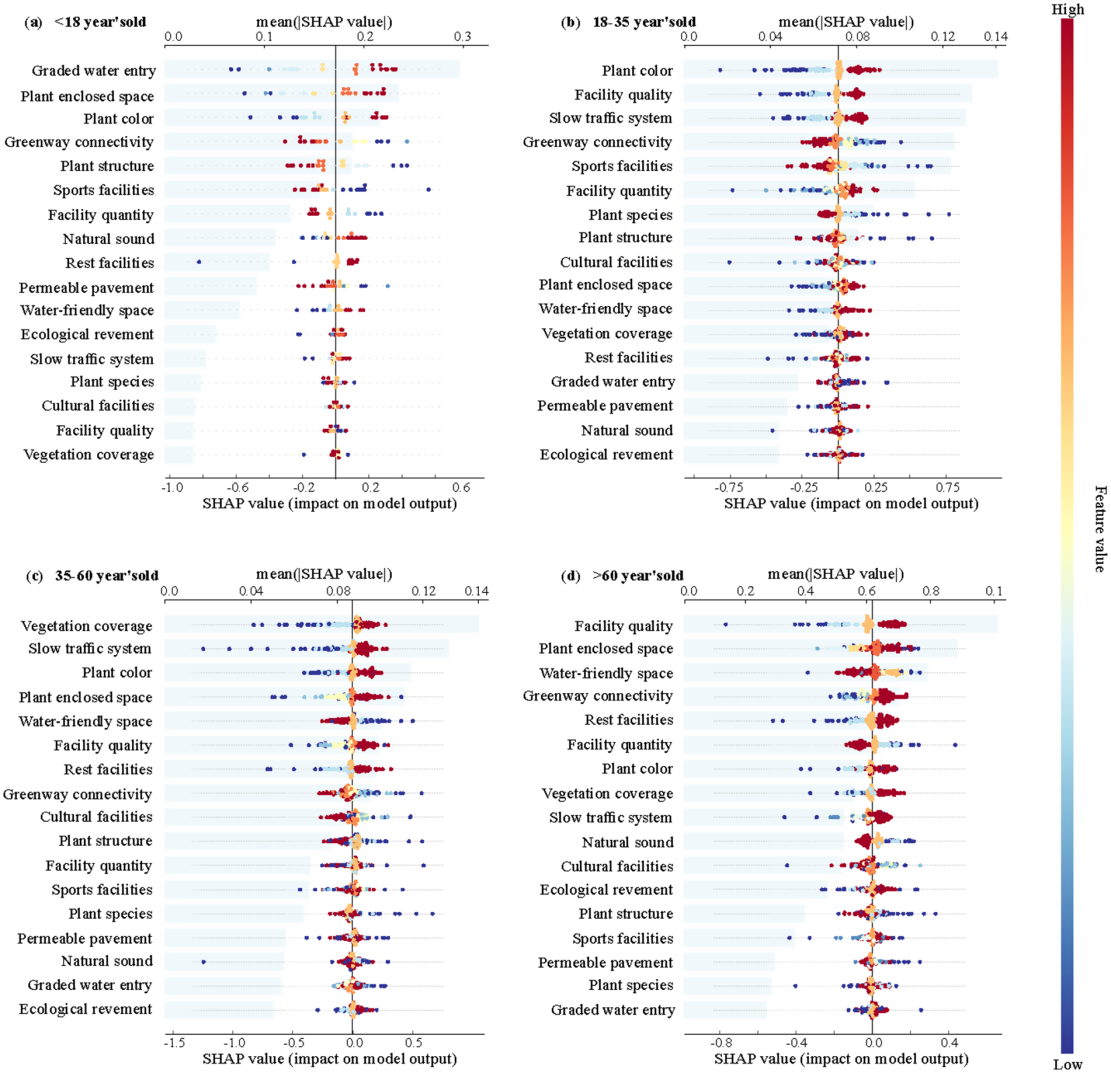
Analysis of key environmental factors affecting perceived recovery in each age group. The Beeswarm plot indicates the distribution status of SHAP values; the bar chart indicates the order of importance of each indicator on perceived recovery. **(a)** < 18 year’s old; **(b)** 18–35 year’s old; **(c)** 35–60 year’s old; **(d)** >60 year’s old.

The results showed (Figure 8) that plant color have high SHAP values in all three age groups (0–18 years, 18–35 years, 35–60 years), indicating a generally positive effect on perceived restorativeness. However, there were significant differences in the sensitivity of different age groups to other landscape elements: the children’s group (0–18 years old) responded more sensitively to Graded water entry and to Vegetation factors (Figure 8a); the quality of facilities and Slow traffic system showed significantly higher SHAP values in the youth group (18–35 years old) (Figure 8b); and the middle-aged group (35–55 years old) showed stronger preferences for Vegetation coverage, Slow traffic system, and Plant color; while the older group (60 years old and above) placed more importance on the quality of facilities and the safety of Water-friendly space (Figure 8d).

#### 3.3.2 Analysis of influencing factors of each restoration dimension

**Figure 9:**
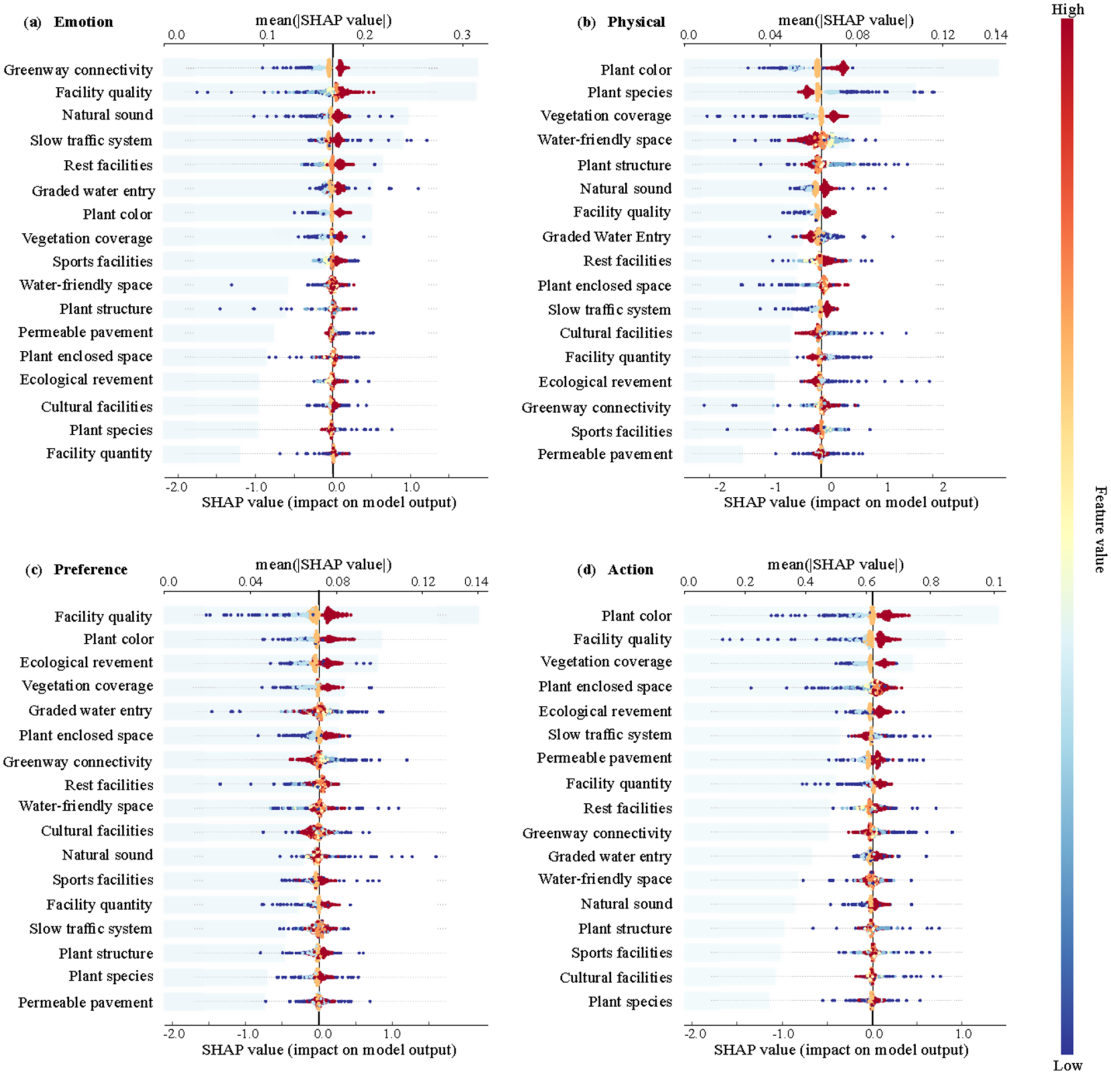
Analysis of key environmental factors affecting the degree of perceived recovery of each sub-dimension of the using population. The Beeswarm plot indicates the distribution status of SHAP values; the bar chart indicates the order of importance of each indicator on perceived recovery. **(a)** Emotion; **(b)** Physical; **(c)** Preference; **(d)** Action.

This study further developed a perceptual restoration model for each sub-dimension of recovery: Emotion, Physical, Preference, and Action. By analyzing SHAP Beeswarm plots of various restoration dimensions and landscape factors, significant differences were identified across the different dimensions of restoration (Figure 9). Specifically, the analysis of these four recovery dimensions and their corresponding landscape factors revealed the following key findings:

In the emotion recovery dimension, the SHAP analysis highlighted Greenway connectivity, Facility quality, and Natural sounds as the most influential landscape features (Figure 9a). Among these, Greenway connectivity contributed the most significantly to the model’s positive output. In the Physical recovery dimension, the analysis showed that Plant color, Plant species, and Vegetation coverage had the greatest impact on the model’s predictions (Figure 9b). Notably, Plant color had the most pronounced positive effect on physiological recovery, with bright and colorful plant configurations notably enhancing the sense of physiological restoration. In contrast, Plant species had an inhibitory effect, suggesting that an overabundance of plant varieties may negatively impact physiological stimulation. For the Preference recovery dimension, SHAP analysis indicated that Facility quality, Plant color, and Ecological revement were the main factors influencing preference recovery (Figure 9c). Lastly, in the Action recovery dimension, the most significant factors were Plant color, Facility quality, and Vegetation coverage (Figure 9d).

#### 3.3.3 Key design factors affecting perceived restorativeness of urban riverside greenways

**Figure 10:**
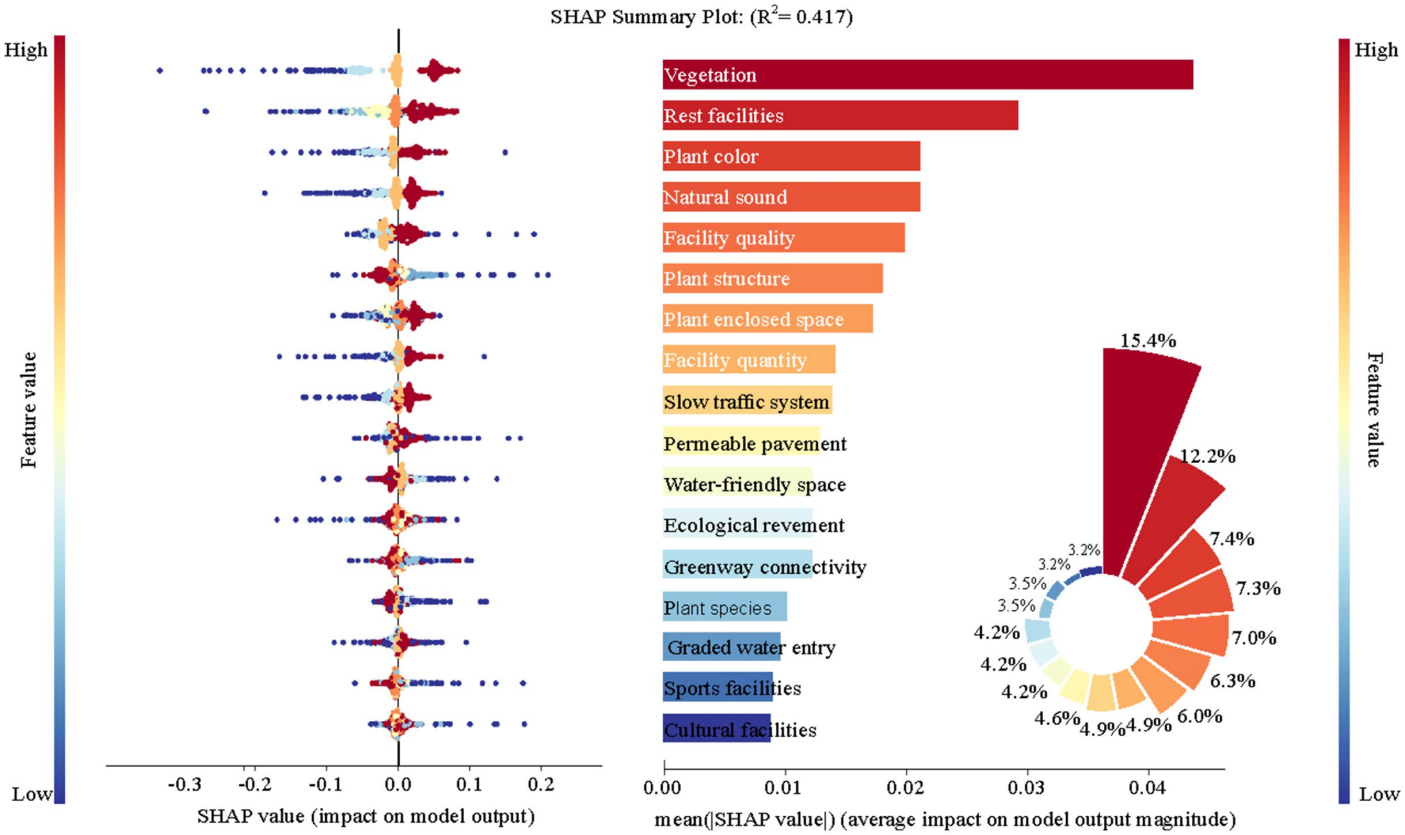
Analysis of key influencing factors affecting the perceived restorativeness of urban riverside greenway users. The horizontal axis in the honeycomb plot represents the SHAP values, and the vertical axis shows the influencing factors, arranged in descending order of their explanatory importance. The bar charts indicate the absolute value importance ranking of each factor for perceived recovery. The relative importance of each factor is expressed as a percentage in the rose diagram.

In the multilayer perceptual machine model analysis of the 17 design factors, the *R*^2^ value of the regression model was 0.417, indicating that the 17 variables explained 41.7% of the variance in perceived restorativeness of urban riverside greenways. Figure 10 illustrates the distribution of SHAP values for each factor. Each point in the figure represents the SHAP value of a feature on a sample, the horizontal axis indicates the size of the SHAP value. The color of each data point represents the magnitude of the corresponding predictor, with red indicating larger eigenvalues and blue indicating smaller eigenvalues. The results show that there are 10 positive and 7 negative indicators that affect the perceived restoration effect of urban riverside greenways.

Among the 17 design factors, Vegetation coverage (A2), Rest facility (D1), Plant color (A1), Natural sounds (B5), Facility quality (D4), and Plant structure (A4) are identified as having relatively higher importance (above 50%). This indicates that these factors are key design elements influencing the perceived restorative benefits of urban riverside greenways. Specifically, Vegetation coverage (A2) and Rest facility (D1) have the highest relative importance, accounting for 15.4 and 12.2%, respectively. These are the most critical design factors. Additionally, Plant color (A1), Natural sounds (B5), and Facility quality (D4) all have a significant positive impact on restorative capacity. In contrast, plant community structure, which is considered a negatively impactful indicator, holds a relative importance of 6.3%.

### 3.4 Influence mechanism analysis

**Figure 11:**
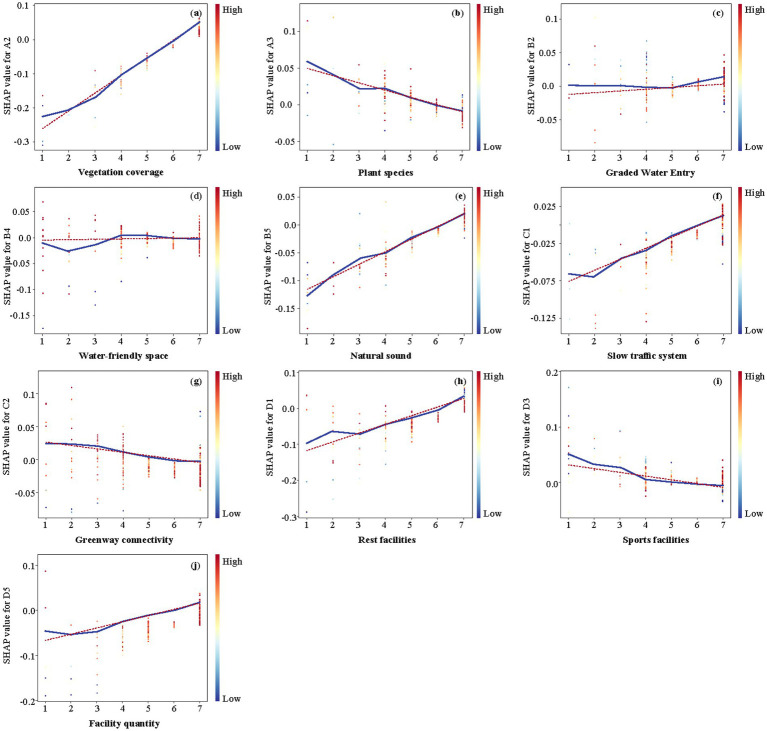
Linear relationship between individual factors on perceived recovery of urban riverside greenways. *x*- and *y*-axes represent individual factors and recovery scores, respectively. Each subplot illustrates the specific contribution of a particular design factor to perceived recovery. Dependency plots illustrate whether the relationship between objectives and features is linear, monotonic, or complex. The variables are ranked in order of their relative importance. The curve represents a LOWESS nonparametric smoothing trendline based on SHAP values. **(a)** Vegetation coverage; **(b)** Plant species; **(c)** Graded water entry; **(d)** Water-friendly space; **(e)** Nature sound; **(f)** Slow traffic sound; **(g)** Greenway connectivity; **(h)** Rest facilities; **(i)** Sports facilities; **(j)** Facility quantity.

**Figure 12:**
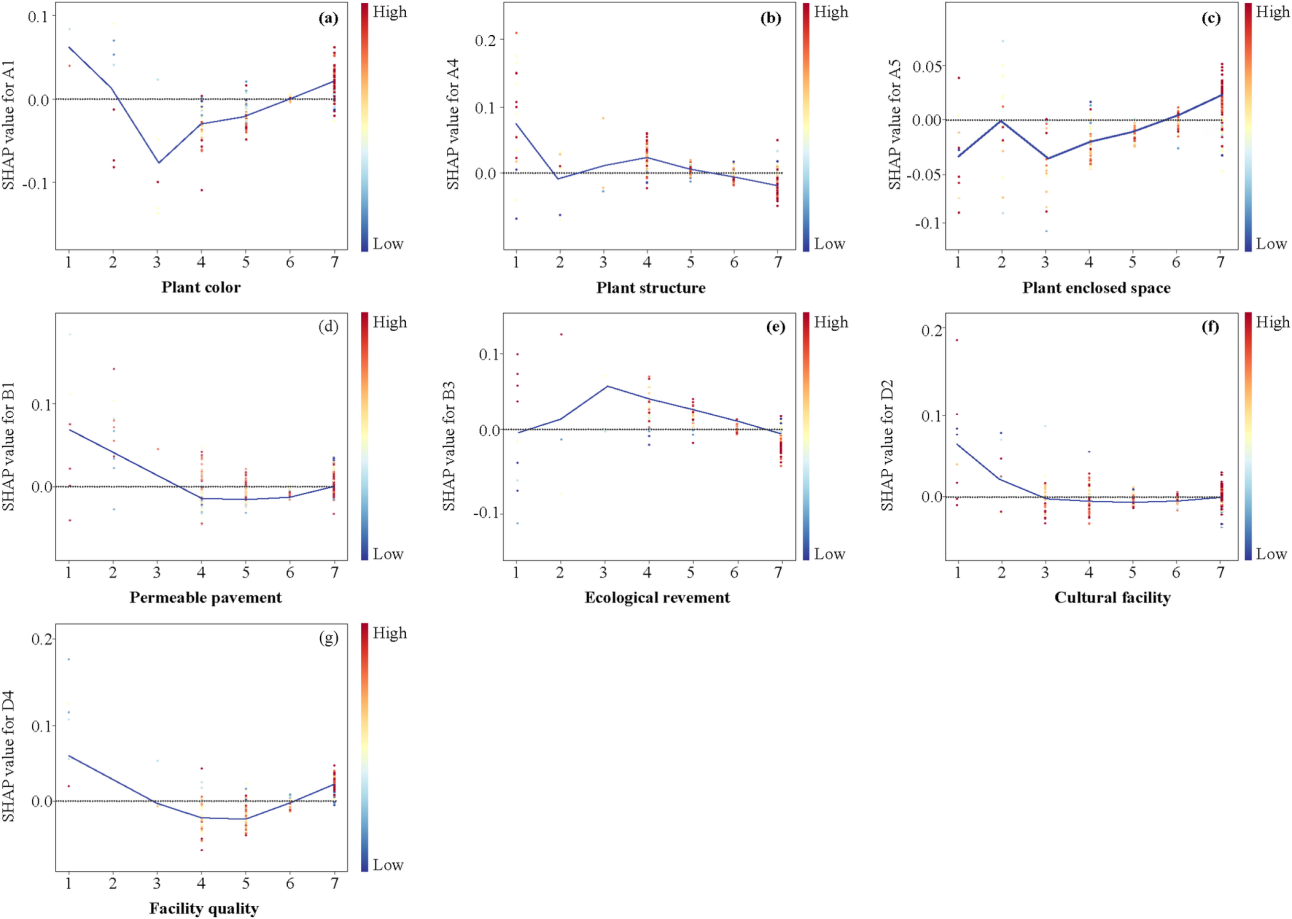
Non-linear relationship between single factors affecting perceived restoration of urban riverside greenways. The curve represents a LOWESS nonparametric smoothing trendline based on SHAP values. **(a)** Plant color; **(b)** Plant structure; **(c)** Plant enclosed space; **(d)** Permeable pavement; **(e)** Ecological revement; **(f)** Cultural facility; **(g)** Facility quality.

To further explore the mechanisms and threshold effects between urban riverside greenway design factors and perceptual restoration ability, dependency plots (Figures 11, 12) were created. The results show that the impact of design factors on perceptual restoration follows both an approximate linear relationship and significant nonlinear relationships, with some factors exhibiting threshold effects. For design factors with trends close to linear, linear regression was used for validation. The results indicated that, although the dependency plot might show slight fluctuations, the overall trend could be described using a linear model, and the linear regression fitting results were statistically significant. Furthermore, no abrupt changes in perceptual restoration values at specific design factor values (i.e., clear threshold points) were observed. Therefore, based on the trends in the dependency plots and the results of the linear regression validation, these factors were classified as linear relationships. Ultimately, 10 linear relationships and 7 nonlinear relationships between design factors and perceptual restoration were identified.

#### 3.4.1 Linear relationships

The results show (Figure 11) that seven design factors, namely Vegetation coverage (A2), Graded water entry (B2), Water-friendly space (B4), Natural sound (B5), Slow traffic system (C1), Rest facility (D1), and Facility quantity (D5), have positive linear relationships with the influence of perceived restoration. The three design factors of Plant species (A3), Greenway connectivity (C2), and Sports facilities (D3) had a negative linear relationship.

#### 3.4.2 Non-linear relationships

The results (Figure 12) indicate that Plant color (A1), Plant structure (A4), Plant enclosed space (A5), Permeable pavement (B1), Ecological revement (B3), Cultural facility (D2), and Facility quality (D4) exhibit complex, non-linear dose–response relationships and threshold effects on the perceptual restorability effects of urban riverside greenways. Specifically, these factors can be categorized into ‘power function-type’ curves, and ‘U-type’ or ‘inverse U-type’ curves (Jiang et al., 2025).

U-type or inverse U-type relationships: Plant color (A1), Permeable pavement (B1), and Facility quality (D4) all show U-shaped relationships with perceptual restorability effects. This suggests that plant color richness and facility comfort have the worst perceptual recovery effects at a “medium” level. In contrast, recovery is improved when the color is either monochromatic or highly varied. Similarly, Permeable pavement (B1) is most effective when permeability is either very low or very high, with the poorest perceptual recovery occurring at a “medium” permeability level. Facility quality (D4) is least effective at the “medium” level, showing the worst recovery at this point. Ecological revement (B3) displayed an inverse U-shape, indicating that recovery is most effective at a semi-artificial, semi-natural “medium” level of ecological intervention.

Power function curves: The relationship between Plant structure (A4) and perceptual restorability effects was overall negative, showing that recovery is best when the community structure is simple (at the ‘very weak’ level). As the complexity of the structure increases, recovery declines sharply, slightly increases at the “weak” level, and then gradually decreases at the “medium” level. For Plant enclosed space (A5), perceptual recovery remains relatively stable from the ‘very weak’ to ‘weak’ levels of enclosure. However, recovery gradually improves as enclosure increases beyond this point. Cultural facilities (D2) show a slow decline in perceived recovery from the ‘very weak’ to ‘moderate’ levels of richness. After reaching a moderate level, the effect on perceptual recovery becomes relatively stable, with no significant changes observed as facility richness increases further.

## 4 Discussion

### 4.1 Perceived recovery benefits of urban riverside greenways before and after use

In environmental psychology research, differences in individual background characteristics may act as covariates that confound the relationship between environmental variables and psychological outcomes. This study confirmed through chi-square test results (Supplementary Table 6) that the “entry” and “exit” groups exhibited high homogeneity in demographic characteristics such as gender and age, demonstrating that large sample sizes can mitigate the feasibility of group inconsistency before and after experiments. This experimental design approach aligns with the methodology adopted by Gascon et al. (2016) in their review of longitudinal studies, which employed large-sample cross-group comparisons to mitigate noise from individual heterogeneity.

This study further reveals that the “leaving” group exhibited a significant improvement in overall perceived recovery after using the greenway (Figure 5). This finding directly addresses the research question of whether greenway environmental experiences yield psychological restorative benefits. It not only quantifies the psychological restorative potential of riverside greenways but also supports the applicability of Attention Restoration Theory (ART) within urban riverside greenway spaces. Xie et al. (2022) previously proposed that greenways effectively alleviate cognitive fatigue by increasing green space exposure. This study further confirms that riverside greenway environments promote perceived recovery across emotional, physiological, preference, and behavioral sub-dimensions. It exhibits particular advantages in physiological perceived recovery, aligning with Van Den Bosch and Ode Sang’s (2017) findings that exposure to natural environments significantly reduces physiological stress indicators like heart rate, blood pressure, and cortisol. This may stem from the close relationship between physiological recovery and improvements in other dimensions, especially emotional and preference recovery. Enhanced emotional and preference states may prompt more positive psychological responses, thereby indirectly promoting physiological system recovery (Ulrich et al., 1991).

### 4.2 Synergistic effects of multidimensional design dimensions on the perceptual restorability effects in urban riverside greenways

Urban green space development has long faced the challenge of balancing ecological preservation with human usage needs (Jalkanen et al., 2025; Xia et al., 2025), creating a mismatch that makes it difficult to balance ecological and human health. In recent years, many scholars have proposed the concept of “multidimensional integration” to create a “win-win” scenario for both ecology and human health (Xia et al., 2025). This approach, which emphasizes “increased synergies and reduced trade-offs,” aims to resolve the conflict caused by the mismatch of demands. However, most of these studies remain conceptual and lack a clear mechanism for achieving synergy (Fan et al., 2025; Yue and Qian, 2022). Our findings indicate that relying solely on ecological design had limited, and in some cases, even negative effects on perceptual restoration (Figure 5). However, when ecological design was integrated synergistically with plant and facility design, the perceived restoration effect was significantly enhanced. This synergistic integration not only produced stronger results but also demonstrated the potential to drive systemic improvement. These findings suggest a positive correlation between ecological health and human health, confirming the existence of a synergistic mechanism that benefits both. These results suggest a positive correlation between ecological and human health outcomes—a relationship that becomes stronger when multidimensional design strategies are implemented cohesively. This provides empirical support for the presence of a synergistic mechanism, reinforcing the potential for holistic design approaches to deliver both environmental and psychological benefits in urban greenway planning.

The study results demonstrate that “The more ecological design does not necessarily mean better perceptual recovery.” The key to designing riverside greenway landscapes lies not in the “quantitative accumulation” of various design dimensions, but in the “optimization of the structure.” This approach is essential for enhancing perceptual restorability effects. While ecological design is a technique aimed at improving the greenway’s ecological environment, it does not directly impact perceptual restorability effects. Rather, it indirectly promotes perceptual restoration by creating a favorable ecological environment (Hung, 2025). The combination of “Plants + Ecology + Facilities” significantly outperforms any single or dual-element combinations in terms of restorative effects, showcasing a clear synergistic relationship. Such synergistic effects have been observed in similar studies. For example, Li et al. (2024) proposed collaborative approaches that combine landscape features and ecological methods to resolve the “esthetic-ecological” conflict. Similarly, in the realm of urban planning, Wei and Yang (2024) suggested that built environment elements have a synergistic effect on residents’ walking experiences. These studies affirm the potential and viability of multidimensional synergies. The mechanism demonstrated here aligns with the “win-win green space planning” concept proposed by Jalkanen et al. (2025), which advocates for balancing ecological health and human well-being through thoughtful landscape design practices. This study’s contribution is twofold: it not only validates specific design combinations but also uncovers synergy as the key mediating mechanism that reconciles conflicting goals. This offers a valuable theoretical foundation and a practical framework for future urban green space design.

### 4.3 Key landscape design factors affecting perceived restoration

Study findings reveal that Vegetation coverage (A2) and Plant color (A1) have significant positive effects on perceived restorativeness. This suggests that, compared to interactions with purely physical urban environments, human engagement with vegetated spaces is more effective in enhancing perceptual restoration (Wang et al., 2016). Specifically, Vegetation coverage (A2) plays a critical role. This finding aligns with the majority of previous studies, which indicate that higher vegetation coverage increases green exposure, thereby significantly promoting perceived restorativeness (Jin et al., 2024; Ramanpong et al., 2024). Likewise, the richness of plant colors contributes to visual pleasure and enhances user preference, which in turn fosters perceptual restoration (Hao et al., 2024a). Plant community structure (A4) exerts a negative influence on perceived recovery, likely because complex plant community structures, despite their ecological value, may induce discomfort in people due to high canopy closure (Hao et al., 2024b). Natural sounds (B5)—such as birdsong and rustling leaves—also significantly enhance perceptual experience along greenways, contributing positively to restoration. Notably, Rest facilities (D1) and Facility quality (D4) emerged as key determinants of perceptual recovery in riverside greenways. This contrasts with some previous research. For instance, Chen and Yang (2024) and Cao et al. (2024) found that Rest facility in urban pocket parks and small green spaces did not significantly promote perceptual recovery. However, other scholars have suggested that the comfort of such facilities in park settings can positively impact restoration (Xu et al., 2024). This discrepancy may stem from differences in spatial typologies. As a linear landscape form, riverside greenways offer distinct waterfront esthetics and visual openness that encourage prolonged stays and scenic engagement (Si et al., 2024). In such environments, users are more inclined to utilize rest facilities for relaxation and mental recovery. Therefore, the comfort and quality of rest facilities not only meet basic physiological needs but also enhance visual accessibility and emotional experiences. These factors indirectly support perceptual restoration, underscoring the crucial role of rest infrastructure in shaping restorative environments along riverside greenways.

### 4.4 Non-linear dose–response relationships between landscape design factors and perceived restorativeness and their group differences

The study finds that Plant color (A1), Plant structure (A4), and Plant enclosed space (A5) are all associated with perceptual recovery in a nonlinear fashion (Figures 11a–c). Specifically, Plant color demonstrates a U-shaped relationship with perceptual recovery, with moderate levels of color richness resulting in the least beneficial effects. This result aligns with literature suggesting that richer plant color diversity is generally more conducive to perceptual recovery. However, the findings of Hao et al. (2024a) contradict this, arguing that perceptual recovery can even be more effective in a uniform green environment (Hoyle et al., 2017). Our results corroborate both perspectives, demonstrating that either a single green vegetation environment or a highly diverse plant color setting can effectively enhance perceptual recovery. The observed relationship between Plant structure (A4) and Plant enclosed space (A5) with perceptual recovery follows a power-function curve. This finding is consistent with the conclusions of Van Den Bogerd et al. (2020a), who posited that simpler plant community structures are more restorative for most people due to their lower cognitive load. Whereas higher plant enclosure leads to a more significant recovery effect, indicating that enclosed vegetation configurations can provide more notable physiological and psychological benefits (Jin et al., 2024).

Further analysis reveals that demographic factors such as gender, age, risk perception levels, and educational background lead to significant differences in the perception of the same landscape elements. These differences not only influence the form of the nonlinear relationship but also substantially affect the actual recovery outcomes across various groups in different spaces. Specifically, women, children, and the elderly tend to prefer spaces with higher plant enclosure. This preference may be due to these groups being more sensitive to risk and physiologically and psychologically relying on environments that provide a greater sense of safety and protection (Weber et al., 2014; Yin et al., 2025). For younger populations, plant color is especially important. Su et al. (2025) suggest that young people are more responsive to color diversity and visual stimuli, which help them achieve psychological relaxation and emotional uplift. Additionally, women are typically more sensitive than men to the complexity of plant color perception and memory. This heightened sensitivity may be linked to an evolutionary tendency to pay closer attention to environmental details. As a result, women tend to experience better recovery in colorful environments. Additionally, people with different educational backgrounds exhibit distinct preferences for plant elements. Those with moderate education levels tend to prefer spaces with simpler community structures, while individuals with higher education levels are more inclined to favor landscapes with diverse community structures or lower plant enclosure. This finding is consistent with the research of Van Den Bogerd et al. (2020a), which shows that higher-educated groups often prefer landscapes that resemble natural, wild states. This reflects their deeper understanding and preference for nature and wilderness esthetics.

In ecofunctional design, Ecological revement (B3) showed an inverted U-shaped relationship with perceptual recovery (Figure 11d), with the optimal threshold for perceptual recovery being reached when the barge was at the “medium” level of semi-artificial and semi-natural, a pattern that has been confirmed in several studies (Bonthoux et al., 2024; Hu et al., 2022). This pattern has been confirmed by several studies (Hu et al., 2022; Tan et al., 2024). In particular, Bonthoux et al. (2024) it is clear that people may prefer ecologically moderate revement designs—a “moderately natural” barge reflects a dynamic balance between clean appearance and ecology(Fischer et al., 2020; Weber et al., 2014). Additionally, Permeable pavement (B1) exhibits a U-shaped relationship with perceived restoration (Figure 11d), indicating that perceived restoration is least effective when the permeability of the paving is at a “moderate” level. This finding contrasts with existing research, such as Zhao et al. (2024) found that the type of paving material did not influence perceived restoration in their study of park pavings. In contrast, this study reveals that the quality or type of paving is not positively correlated with perceived restoration. This discrepancy may arise from the public’s limited understanding of the ecological function of permeable pavement, which often leads to its neglect. Such a gap in conceptual understanding could weaken users’ environmental identity towards ecological designs, causing permeable pavement to be perceived merely as “ordinary pavement” on a sensory level, thus failing to trigger psychological restoration mechanisms based on ecological value.

In the design of service functions, Facility quality (D4) has a U-shaped relationship with perceived recovery (Figure 11g), i.e., facilities of moderate quality correspond to the lowest perceived recovery benefits. This phenomenon may stem from differences in the needs of different populations. Children and low-education groups were less concerned with Facility quality (D4), and Chen et al. (2025) noted that the number of facilities and the richness of play options were much more important to children’s perceived recovery than the quality of a single facility. Lower educated groups, on the other hand, are more concerned with facility utility, such as infrastructure like seating, toilets, and bins (Van Den Bogerd et al., 2020b). In contrast, younger adults, the elderly, and individuals with higher education levels have a greater demand for high-quality facilities, though their motivations differ. Elderly people are more likely to use greenways for socializing and resting, often valuing comfort and relaxation features of facilities. According to Rodiek and Fried (2005), elderly individuals tend to prioritize the comfort of resting facilities and the tranquility of the surrounding environment. Meanwhile, younger adults and highly educated individuals prefer well-designed, multifunctional, and sustainable facilities (Huang et al., 2020). This may be due to their higher stress levels a space for mental relaxation and stress relief (Sun and Jiang, 2025). Additionally, the diversity of Cultural facilities (D2) has a diminishing effect on perceived restoration as the variety increases (Figure 11f). This suggests that a wide range of cultural facilities may not necessarily promote perceived restoration. This may be because an excessive accumulation of cultural facility types can lead to information overload, distracting users and ultimately hindering the formation of a deep restorative experience.

Overall, the findings of this study suggest that the relationship between riverside greenway design factors and perceived restoration is not a simple linear pattern. Instead, it follows a diverse, nonlinear dose–response relationship, including U-shaped, inverted U-shaped, and power-function forms, among others. This complexity reflects that the role of landscape elements in perceived restoration is not always “more is better” or “the more natural, the better.” Rather, it is influenced by the interaction of multiple mechanisms, such as visual, psychological, and ecological cognition. Moreover, this nonlinear relationship may arise not only from the design factors themselves but also from the complexity of individual user needs. This further underscores the need for landscape designs that can accommodate the diversity of riverside greenway users. A more heterogeneous landscape is more likely to meet the needs of a varied visitor population (Hao et al., 2024a). This strategy not only enhances the overall benefits of the landscape but also provides a feasible approach for the sustainable planning of urban waterfront spaces.

### 4.5 Limitations and future research

This study focuses on the riverside greenway along the Hangzhou section of the Beijing-Hangzhou Grand Canal. Although this area possesses unique ecological and cultural characteristics, the environmental features of the Grand Canal greenway cannot represent all types of urban riverside greenways. Therefore, the applicability of the research conclusions may be constrained by regional characteristics and cannot be universally applied to other types of greenways, particularly those associated with different geographical environments, climatic conditions, or cultural contexts. Future research could validate the universality of these findings through comparative analyses across multiple regions and greenway types. Additionally, systematic multi-seasonal tracking or cross-seasonal comparative experiments should be conducted to examine the varying impacts of seasonal changes on perceived restorative benefits. Furthermore, in terms of experimental design, this study primarily relied on field observations and cross-sectional data analysis, leaving room for improvement in rigorous causal inference. During questionnaire data collection, the study lacked consistency control between pre- and post-greenway usage groups. Although the large sample size partially mitigates this limitation, it may still influence the findings. Furthermore, given the complexity of human perceptual recovery, future research could adopt panel data tracking designs to establish long-term longitudinal observation mechanisms, thereby capturing the sustained impacts of greenway environments on physical and mental health. Additionally, multifunctional urban greenway studies should account for differing design element requirements across various usage purposes.

## 5 Conclusion

This study focuses on the riverside greenway along the Hangzhou section of the Grand Canal, developing an evaluation framework comprising four design dimensions and 17 indicators. By leveraging the high accuracy of machine learning models, it delves into the impact mechanisms of design factors on perceived restoration in urban riverside greenways. The findings reveal that design factors have a complex impact on perceived restoration, which in turn influences psychological well-being. Specifically, six features were identified that affect perceived restoration in the landscape design of urban riverside greenways. Furthermore, the study uncovered both linear and nonlinear dose–response relationships between these design factors and perceived restoration. For instance, Plant color, Permeable pavement, and Facility quality exhibit a U-shaped relationship, while ecological revetment show an inverted U-shaped relationship. Interestingly, these design factors have different levels of impact on diverse groups, with significant variations based on age, gender, and cultural background. Respondents from different demographic categories demonstrated diverse needs when interacting with heterogeneous landscapes. From a design perspective, this research highlights that the synergistic integration of “Plant + Ecology + Facility” is an effective strategy for achieving a win-win scenario in both landscape ecological health and human health. These findings provide scientific evidence for creating restorative environments in urban riverside greenways and offer solid theoretical and practical guidance for future landscape design that balances both ecological and human health outcomes.

## Data Availability

The raw data supporting the conclusions of this article will be made available by the authors, without undue reservation.
