# Supplementary material for: Bridging landscape and perceived restorativeness: an empirical study of greenways along the Grand Canal, Hangzhou
Source: Front Psychol. 2026 Feb 5;17:1742799. doi: 10.3389/fpsyg.2026.1742799 (PMC12916418; doi:10.3389/fpsyg.2026.1742799)
Supplement: Supplementary file 1 [file Table_1.docx]

Supplementary Material

**This file includes:**

**Supplementary tables**

**Supplementary Table1.** Indicators for evaluating the environmental resilience of urban riverfront greenways

**Supplementary Table2.** Short Revision Rating Scale (SRRS) — Entering Crowd

**Supplementary Table3.** Short Revision Rating Scale (SRRS) — Leaving Crowd

**Supplementary Table4.** Variance Inflation Factor (VIF) Diagnostic Analysis.

**Supplementary Table5.** MLP Regression Model Hyperparameter Specifications.

**Supplementary Table6.**Demographic Characteristics and Group Comparisons (Chi-square Tests, N=1052)

**Supplementary Table7.**Results of Two-Way ANOVA on Demographics

**Supplementary Table8.**Comparison of Scores Between Groups before and after respondents' use of the greenway Using the Mann–Whitney U Test

**Supplementary figures**

**Supplementary Fig. 1** Photographs Illustrating the Spatial Structure of the Greenway.

**Supplementary Fig. 2** Photographs Illustrating the Winter Vegetation and Landscape of the Greenway

**Supplementary notes:**

Detailed Description of Questionnaire Design and Experiment

**Supplementary Table1.** Indicators for evaluating the environmental resilience of urban riverfront greenways

| **Criterion layer** | **Indicator** | **Definition** | **Quantitative standard** | **Question** |
| --- | --- | --- | --- | --- |
| Plant Design(A) | Plant color(A1) | Plant colours presented in the greenway landscape.single colour, which denotes green as the basic hue, and multiple colours, which include several colours containing green  (Hao et al., 2024) | 1 = A single green color,  4 = 1–2 dominant plant colors,  7 = More than three dominant plant colors. | The rich colours of the plants in the greenway help me recover from mental fatigue. |
|  | Vegetation coverage  (A2) | Percentage of the ground area covered by the vertical projection of vegetation (including leaves, stems and branches) in relation to the area of the surveyed area.  Peschardt etal. (2016)  Nordh etal. (2009) | 1 = 0–40%, 4 = 40–70%, 7 = 70% and above. | The extensive vegetation cover in the greenway brings me a sense of pleasure. |
|  | Plant species  (A3) | Number of plant species observed within the greenway landscape.Fang et al. (2009) | 1 = Plant communities appear similar, composed of a few species, 4 = Contain several typical street tree species, 7 = Plant communities are species-rich and include varied forms of vegetation. | The rich variety of plants in the greenway helped me recover from mental fatigue. |
|  | Plant structure  (A4) | The plant community is vertically stratified and includes a tree layer, a shrub layer, an herb layer and a Herbaceous Layer.  (Da et al., 2024) | 1 = tree layer +Herbaceous Layer  4 = tree layer +shrub layer+Herbaceous Layer,  7 =Large tree layer + Small Tree layer+shrub layer+Herbaceous Layer. | The rich flora of a greenway can relax me. |
|  | Plant enclosed space(A5) | Degree of enclosure in the lower and middle levels of the plant space, the ratio of the portion of the boundary of the plant space where small trees and shrubs overlap (L1) to the perimeter of the boundary (L).Peng (2018) | 1 = Less than 30% open space,  4 = 30–70% semi-enclosed space,  7 = More than 70% plant-enclosed or fully enclosed space. | Having a relatively large number of plants enclosing spaces in the greenway helps me recover from mental fatigue. |
| Ecological Design(B) | Permeable pavement  (B1) | The permeability of the pavement used in the greenway.  Guo et al.（2020） | 1 = Almost impermeable (hardscape pavement),  4 = Moderately permeable (combination of permeable and hard pavement),  7 = Highly permeable natural pavement. | The lack of standing water in the greenway was conducive to my relaxation. |
|  | Graded water entry  (B2) | Providing visitors with a richer hierarchy of views, inner test visitors will gain a sense of guidance and belonging, while outer visitors will still be able to view the waterscape from a longer distance.  (Fei et al., 2023) | 1 = Vertical water entry,  4 = Natural gentle slope or stepped water entry,  7 = Land level with water surface. | The fact that the green road surface is higher than the riverbank line is conducive to my relaxation. |
|  | Ecological revement  (B3) | Shore protection measures are of the type of natural slopes, willow and cedar piles, as well as ecological gabions, ecological retaining walls and ecological berms.Ma et al.(2022) | 1 = Completely Artificial,  4 = Semi-Artificial and Semi-Natural,  7 = Completely Natural. | The greenway river is a vibrant natural scene, which is much more relaxing for me. |
|  | Water-friendly space(B4) | The extent to which the edges of water bodies provide safe,comfortable, and accessible spaces or facilities for the public to approach and stay. 8.The slope of the shoreline and the accessibility of the water body to people(Wang et al., 2024) | 1 = No water-friendly facilities,  4 = Water-friendly facilities with limited or restricted spaces,  7 = Spacious, continuous, safe, and well-maintained water-friendly facilities. | The water-friendly spaces within the greenway area help me recover from mental fatigue. |
|  | Nature sound(B5) | The relative strengthof natural sounds (such as water flow and bird calls )compared to human-made noise in the greenway.  (García-Martín et al., 2025) | 1 = Dominated by artificial sounds,  4 = There are some artificial sounds, 7 = Dominated by rich natural sounds. | The sounds of the natural environment that I hear in the greenway can relax me. |
| Path Design(C) | Slow traffic system(C1) | Whether the greenway space is exclusively for pedestrian and slow use, free from non-motorized vehicles like bicycles, and provides a safe and tranquil environment.  (Wang et al., 2024) | 1 = Mixed with non-motorized vehicles,  4 = Pedestrian and bicycle lanes are independent and separated,  7 = Designated exclusively for pedestrian use. | Greenway trails without non-motorised distractions are much more relaxing for me. |
|  | Greenway connectivity  (C2) | Whether the greenway path is continuous, uninterrupted, and does not require detouring or leaving the greenway due toobstacles..Lei et al.（2020） | 1 = The path has obvious discontinuities or obstacles,  4 = The path is continuous but requires minor detours,  7 = The path is continuous and uninterrupted. | A break in the greenway walk would affect me. |
| Facility Design(D) | Rest facility(D1) | Types of rest facilities such as gazebos, promenades, and seating within the greenway.  Pasha et al.(2013) | 1 = Almost no rest facilities,  4 = Have 2-3 different types,  7 = A rich variety of types with diverse functions. | I prefer to stay longer in areas of the greenway that have rest facilities. |
|  | Cultural facility(D2) | Facilities with cultural functions such as cultural landscape walls, vignettes, and buildings within the greenway.   Xu et al. (2024) | 1 = Almost no cultural facilities,  4 = Have 2-3 different types,  7 = A rich variety of types with diverse functions. | The cultural facilities in the greenway can better help me relax. |
|  | Sports facility(D3) | The variety and number of sports and fitness facilities (Plastic running track, fitness equipment)in the greenway. Xu et al. (2024) | 1 = Almost no sports facilities,  4 = Have 2-3 different types,  7 = A rich variety of types with diverse functions. | The sports facilities in the greenway can better help me relax. |
|  | Facility quality(D4) | The comfort, condition, and overall maintenance level of various facilities within the greenway.Xu et al.(2024) | 1 = Poor quality/poorly maintained,  4 = Average design quality and maintenance,  7 = Excellent design and well-maintained. | The comfort of the facilities in the greenway helped me recover from mental fatigue. |
|  | Facility quantity(D5) | Number of amenities in the greenway.Whether the quantity of various facilities in the greenway is sufficient.Paquet et al.(2013) | 1 = Facilities are extremely scarce,  4 = Facilities are basically sufficient for use,  7 = Facilities are sufficient and convenient. | A larger amount of amenity space helps me with mental fatigue. |

**Supplementary Table2.** Short Revision Rating Scale (SRRS) — Entering Crowd

|  | | | |
| --- | --- | --- | --- |
| How would you describe your emotional state on city roads? | | | |
| V1 | fierce | 1□ 2□ 3□ 4□ 5□ 6□ 7□ | cheerful |
| V2 | scared | 1□ 2□ 3□ 4□ 5□ 6□ 7□ | relaxed |
| How would you describe your physical state on city roads? | | | |
| V3 | I feel fatigued. | 1□ 2□ 3□ 4□ 5□ 6□ 7□ |  |
| V4 | I need more rest. | 1□ 2□ 3□ 4□ 5□ 6□ 7□ |  |
| How would you describe your state of mind when you are in the middle of a city road? | | | |
| V5 | I was intrigued by the scenes I saw along the way. | 1□ 2□ 3□ 4□ 5□ 6□ 7□ |  |
| V6 | I was preoccupied with certain scenes I saw along the way. | 1□ 2□ 3□ 4□ 5□ 6□ 7□ |  |
| How would you describe your behaviour on city roads? | | | |
| V7 | I hope to come here often. | 1□ 2□ 3□ 4□ 5□ 6□ 7□ |  |
| V8 | I wish I could have stayed longer. | 1□ 2□ 3□ 4□ 5□ 6□ 7□ |  |

**Supplementary Table3.** Short Revision Rating Scale (SRRS) — Leaving Crowd

|  | | | |
| --- | --- | --- | --- |
| How would you describe your feelings when you leave the Greenway? | | | |
| V1 | fierce | 1□ 2□ 3□ 4□ 5□ 6□ 7□ | cheerful |
| V2 | scared | 1□ 2□ 3□ 4□ 5□ 6□ 7□ | relaxed |
| How would you describe your physical state when you leave the greenway? | | | |
| V3 | I feel fatigued. | 1□ 2□ 3□ 4□ 5□ 6□ 7□ |  |
| V4 | I need more rest. | 1□ 2□ 3□ 4□ 5□ 6□ 7□ |  |
| How would you describe your mental state when you leave the greenway? | | | |
| V5 | I was intrigued by the scenes I saw along the way. | 1□ 2□ 3□ 4□ 5□ 6□ 7□ |  |
| V6 | I was preoccupied with certain scenes I saw along the way. | 1□ 2□ 3□ 4□ 5□ 6□ 7□ |  |
| How would you describe your behaviour when you leave the Greenway? | | | |
| V7 | I hope to come here often. | 1□ 2□ 3□ 4□ 5□ 6□ 7□ |  |
| V8 | I wish I could have stayed longer. | 1□ 2□ 3□ 4□ 5□ 6□ 7□ |  |

**Supplementary Table4.** Variance Inflation Factor (VIF) Diagnostic Analysis.

| Item/Variable | Variance Inflation Factor (VIF) | Tolerance |
| --- | --- | --- |
| A1 | 2.638 | 0.379 |
| A2 | 3.119 | 0.321 |
| A3 | 2.726 | 0.367 |
| A4 | 1.455 | 0.687 |
| A5 | 1.599 | 0.626 |
| B1 | 1.511 | 0.662 |
| B2 | 1.763 | 0.567 |
| B3 | 1.677 | 0.596 |
| B4 | 1.393 | 0.718 |
| B5 | 1.675 | 0.597 |
| C1 | 1.764 | 0.567 |
| C2 | 1.379 | 0.725 |
| D1 | 1.597 | 0.626 |
| D2 | 1.787 | 0.560 |
| D3 | 1.943 | 0.515 |
| D4 | 2.233 | 0.448 |
| D5 | 1.897 | 0.527 |

**Supplementary Table5.** MLP Regression Model Hyperparameter Specifications.

| Parameter | Value | Parameter | Value |
| --- | --- | --- | --- |
| activation | relu | alpha | 0.0001 |
| hidden_layer_sizes | (100,50) | learning_rate | 0.001 |
| max_iter | 1000 | tol | 1.00E-04 |
| solver | adam | batch_size | 64 |

**Supplementary Table6.** Demographic Characteristics and Group Comparisons (Chi-square Tests, N=1052)

| Variable | Category | GROUP(%) | | Total | χ2 | p |
| --- | --- | --- | --- | --- | --- | --- |
|  |  | 1.0 | 2.0 |  |  |  |
| Gender | Men | 261(49.62) | 256(48.67) | 517(49.14) | 0.095 | 0.758 |
|  | Women | 265(50.38) | 270(51.33) | 535(50.86) |  |  |
| Age | <18 | 36(6.84) | 24(4.56) | 60(5.70) | 4.371 | 0.224 |
|  | 18-35 | 141(26.81) | 146(27.76) | 287(27.28) |  |  |
|  | 35-60 | 199(37.83) | 185(35.17) | 384(36.50) |  |  |
|  | >60 | 150(28.52) | 171(32.51) | 321(30.51) |  |  |
| Education Level | Junior high  and below | 104(19.77) | 111(21.10) | 215(20.44) | 0.295 | 0.961 |
|  | Senior high  And secondary vocational school | 146(27.76) | 144(27.38) | 290(27.57) |  |  |
|  | Associate and Bachelor’s Degrees | 237(45.06) | 232(44.11) | 469(44.58) |  |  |
|  | Postgraduate degree and above | 39(7.41) | 39(7.41) | 78(7.41) |  |  |
| Usage Frequency | Almost everyday | 132(25.10) | 164(31.18) | 296(28.14) | 10.112 | 0.018* |
|  | Twice a week or more | 134(25.48) | 145(27.57) | 279(26.52) |  |  |
|  | Once a week | 74(14.07) | 76(14.45) | 150(14.26) |  |  |
|  | Occasionally | 186(35.36) | 141(26.81) | 327(31.08) |  |  |
| Usage Duration | <30 minutes | 88(16.73) | 92(17.49) | 180(17.11) | 0.675 | 0.879 |
|  | 30-60 minutes | 223(42.40) | 210(39.92) | 433(41.16) |  |  |
|  | 1-2 hours | 166(31.56) | 172(32.70) | 338(32.13) |  |  |
|  | 2 hours and above | 49(9.32) | 52(9.89) | 101(9.60) |  |  |

* p<0.05 ** p<0.01

**Supplementary Table7.** Results of Two-Way ANOVA on Demographics

| Source | Sum of Squares | *df* | Mean Square | *F* | *p* |
| --- | --- | --- | --- | --- | --- |
| Intercept | 2.912 | 1 | 2.912 | 7.753 | 0.006** |
| Duration | 4.068 | 3 | 1.356 | 3.611 | 0.013* |
| Frequency | 7.110 | 3 | 2.370 | 6.310 | 0.000** |
| Duration × Frequency | 3.130 | 9 | 0.348 | 0.926 | 0.502 |
| Gender | 0.119 | 1 | 0.119 | 0.317 | 0.574 |
| Age | 0.695 | 1 | 0.695 | 1.850 | 0.174 |
| Education | 1.514 | 1 | 1.514 | 4.032 | 0.045* |
| Residual | 190.407 | 507 | 0.376 |  |  |

* p<0.05 ** p<0.01

**Supplementary Table8.** Comparison of Scores Between Groups before and after respondents' use of the greenway Using the Mann–Whitney U Test

| Variable | Median (P25, P75) | | Mann–Whitney U | z-value | p |
| --- | --- | --- | --- | --- | --- |
|  | Before(n=526) | After(n=526) |  |  |  |
| Emotion | 5.000(3.5,6.0) | 6.500(6.0,7.0) | 70697.500 | -14.054 | 0.000** |
| Physical | 3.000(1.5,4.0) | 5.500(3.0,6.0) | 68377.500 | -14.366 | 0.000** |
| Preference | 4.500(3.0,6.0) | 6.000(5.0,7.0) | 85478.000 | -10.816 | 0.000** |
| Action | 5.000(2.5,6.0) | 6.000(5.0,7.0) | 79700.000 | -12.034 | 0.000** |
| Overall | -0.392(-1.2,0.3) | 0.617(0.1,1.0) | 55298.500 | -16.855 | 0.000** |

* p<0.05 ** p<0.01

**Supplementary notes:**

**Detailed Description of Questionnaire Design and Experiment**

1.Experimental Conditions: Hangzhou City, Zhejiang Province, China, features a subtropical monsoon climate. According to historical meteorological data provided by the China Meteorological Administration (National Meteorological Information Center, 2023), winter temperatures remain stable, with average highs exceeding 10°C and lows maintaining between 2-3°C. During the period from December 2024 to February 2025, Hangzhou's average temperature ranged from 11°C to 3°C, with a cumulative total of 39 sunny days. This stable and relatively warm climate prevents Hangzhou's winter from presenting a uniformly barren landscape. Field surveys reveal that numerous evergreen plants retain their green foliage, while deciduous plants exhibit rich seasonal coloration (Supplementary Fig. 2). Although deciduous plants display withered leaves, they also offer unique winter ornamental value. Frequent sunny weather further facilitates outdoor activities for Hangzhou residents during winter. The experiment was conducted from December 2024 to January 2025, specifically during sunny periods with cloud cover <30%, temperatures between 5–10°C, Beaufort wind force 2–3, and times from 9:00–11:00 and 13:00–15:00.

2.The survey process consists of three steps: (1) Conduct crowd surveys at 14 high-traffic sample points designated as entry/exit points. Interview respondents: those entering for less than 10 minutes are classified as incoming visitors; those indicating departure or leaving within 10 minutes are classified as outgoing visitors. Existing health recovery studies indicate that residents require a certain amount of time to experience positive effects in green spaces (Tu et al., 2026) and propose a minimum distance of 0.75 kilometers for natural exposure to produce psychophysiological effects (Tu et al., 2026), equating to 9 to 11 minutes. Additionally, the spatial connectivity of our study area is relatively poor, with some users re-entering or turning back. Relying solely on spatial location (entrances/exits) to determine entry/exit status may erroneously classify such “returning” users as “first-time entrants.” Therefore, we selected time as a threshold to distinguish between users who had just arrived at the greenway (exposure time ≤ 10 minutes) and those who had already spent some time browsing (exposure time > 10 minutes).(2) After explaining the study objectives and obtaining informed consent, participants completed the questionnaire. For those completing the post-test questionnaire, researchers provided explanations of the landscape design factor rating scale in relation to the greenway environment to ensure accurate understanding and objective scoring. (3) Researchers reviewed questionnaires to identify omissions.

3. Questionnaire Screening: This study collected a total of 1,160 questionnaires. After data cleaning (excluding invalid data such as contradictory responses and systematic answering patterns), 1,052 valid questionnaires were retained (a validity rate of 90.34%), with 526 each assigned to the entry group and the exit group.

We conducted a rigorous, multi-stage screening of the initial questionnaires based on post-identification methods summarized in existing research (Zhong et al., 2023). Three primary methods were employed: embedded identification scales, response pattern recognition, and reaction time analysis. First, in the initial round, we embedded “trap questions” and “indicator questions” to directly eliminate responses from participants who did not carefully read the questions (Dunn et al., 2018). The second round employed response pattern recognition (reactivity screening). By analyzing metrics such as the long string index and individual response standard deviation, we identified and discarded questionnaires exhibiting linear response patterns or abnormal reaction patterns (Meade et al., 2012). The third round involved reaction time recognition, based on Huang et al. (2012)'s proposal that a reasonable response time per item should be 10-15 seconds. The questionnaire used in this study comprised 25 items, with a theoretical completion time of approximately 4.2–6.3 minutes. Preliminary research indicated that multiple participants completed the questionnaire within 5–8 minutes in real-world settings. Therefore, considering both theoretical and practical response times, we set a minimum response time threshold of 5 minutes. Finally, we excluded questionnaires with completion times under 5minutes—indicating excessively rapid responses—from the raw data.Through these three rounds of rigorous data screening, we ultimately selected 526 questionnaires for both the entry and exit groups.


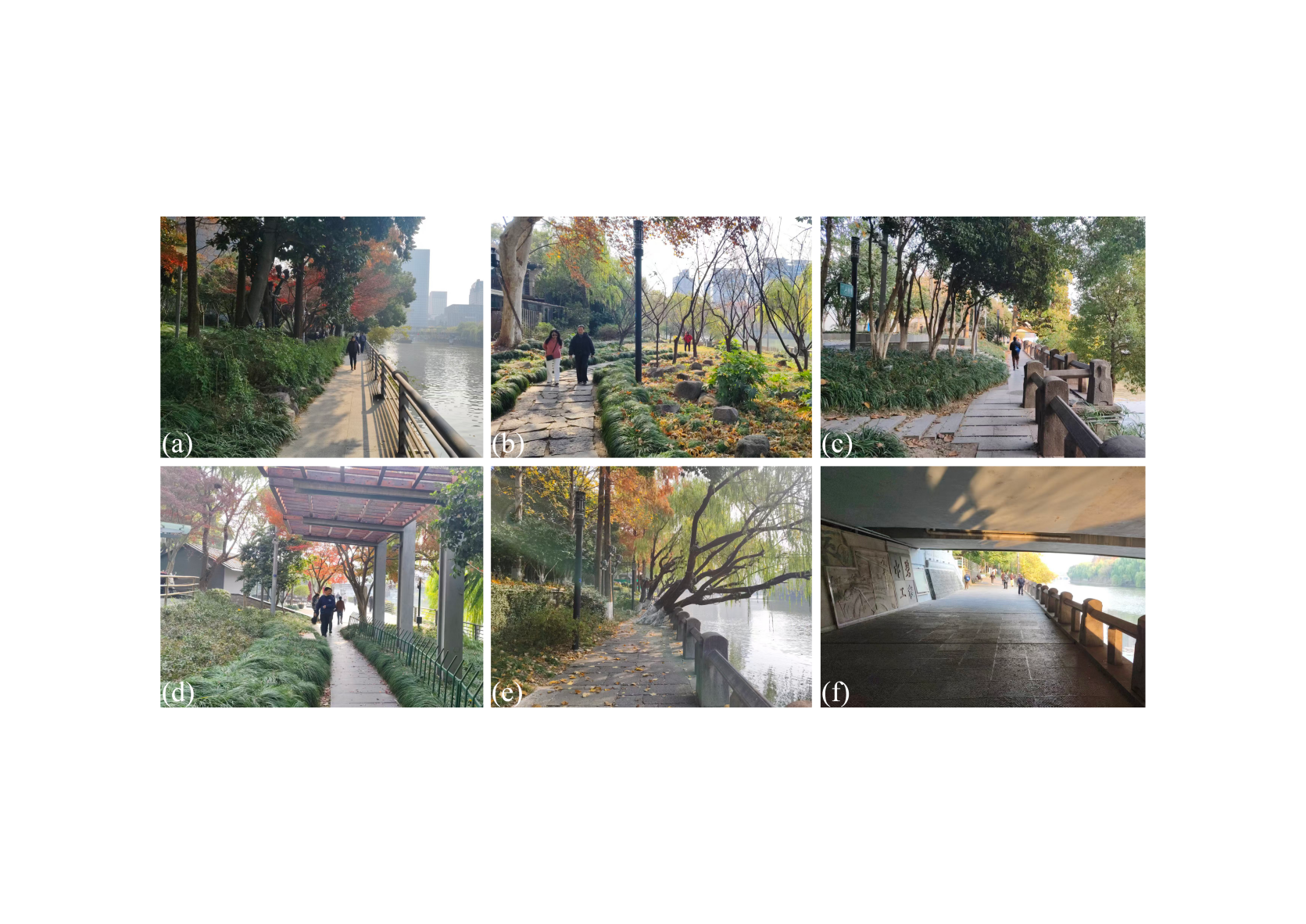


Supplementary Fig. 1 Photographs Illustrating the Spatial Structure of the Greenway.


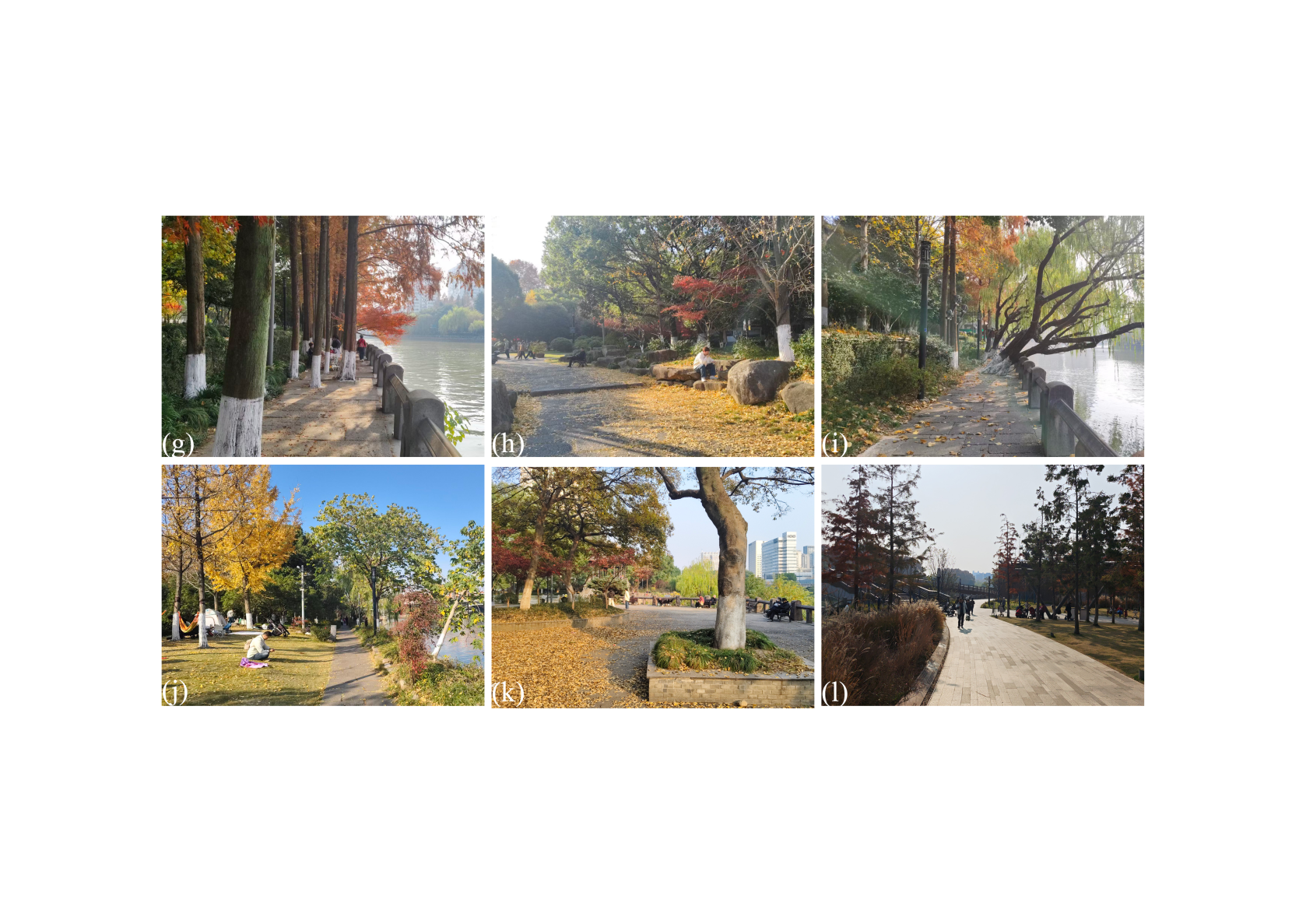


Supplementary Fig. 2 Photographs Illustrating the Winter Vegetation and Landscape of the Greenway
